# Air pollution, genetic factors and the risk of osteoporosis: A prospective study in the UK biobank

**DOI:** 10.3389/fpubh.2023.1119774

**Published:** 2023-03-21

**Authors:** Xing-Hao Yu, Han-Wen Cao, Lin Bo, Shu-Feng Lei, Fei-Yan Deng

**Affiliations:** ^1^School of Public Health, Center for Genetic Epidemiology and Genomics, Medical College of Soochow University, Suzhou, China; ^2^Jiangsu Key Laboratory of Preventive and Translational Medicine for Geriatric Diseases, Soochow University, Jiangsu, China; ^3^Department of Rheumatology, The Second Affiliated Hospital of Soochow University, Suzhou, Jiangsu, China; ^4^Collaborative Innovation Center of Bone and Immunity Between Sihong Hospital and Soochow University, Jiangsu, China

**Keywords:** osteoporosis, air pollution score, genetic risk score, genetic factor, air pollutant

## Abstract

**Purpose:**

To reveal relationship between air pollution exposure and osteoporosis (OP) risk.

**Methods:**

Based on large-scale data from the UK Biobank, we evaluated the relationship between OP risk and several air pollutants. Then air pollution scores (APS) were constructed to assess the combined effects of multiple air pollutants on OP risk. Finally, we constructed a genetic risk score (GRS) based on a large genome-wide association study of femoral neck bone mineral density and assessed whether single or combined exposure to air pollutants modifies the effect of genetic risk on OP and fracture risk.

**Results:**

PM_2.5_, NO_2_, NO_x_, and APS were significantly associated with an increased risk of OP/fracture. OP and fracture risk raised with increasing concentrations of air pollutants: compared to the lowest APS quintile group, subjects in the highest quintile group had a hazard ratio (HR) (95% CI) estimated at 1.140 (1.072–1.213) for OP and 1.080 (1.026–1.136) for fracture. Moreover, participants with low GRS and the highest air pollutant concentration had the highest risk of OP, the HRs (95% CI) of OP were 1.706 (1.483–1.964), 1.658 (1.434–1.916), 1.696 (1.478–1.947), 1.740 (1.506–2.001) and 1.659 (1.442–1.908), respectively, for PM_2.5_, PM_10_, PM_2.5−10_, NO_2_, and NO_x_. Similar results were also observed for fractures. Finally, we assessed the joint effect of APS and GRS on the risk of OP. Participants with higher APS and lower GRS had a higher risk of developing OP. Similar results were observed in the joint effect of GRS and APS on fracture.

**Conclusions:**

We found that exposure to air pollution, individually or jointly, could improve the risk of developing OP and fractures, and increased the risk by interacting with genetic factors.

## 1. Introduction

Osteoporosis (OP) is a systemic bone disease characterized by low bone mineral density (BMD), bone fragility, and devastation of the microstructure of bone tissue, which occurs when bone destruction exceeds new bone formation (1). As the population is aging, OP will develop dramatically, which will cause enormous social and economic stress. As a major cause of public health threats, long-term ambient air pollution exposure is related to the increased risk of complex diseases (e.g., cardiovascular diseases, respiratory diseases, malignant tumors), and increased morbidity and mortality worldwide (2).

Previous studies have shown inconsistent associations between air pollution exposure and the risk of OP and fracture (Supplementary Table S1) (3–12). For example, a study using data from the UK Biobank confirmed that higher air pollution exposure was associated with lower eBMD levels and increased prevalence of osteoporosis (13). A cross-sectional study of 1,039 subjects showed no significant association between BMD with particulate matter (PM) exposure after correcting for age and sex (14), but some studies have reported an association of PM with an aerodynamic diameter ≤ 2.5 μm (PM_2.5_) and PM with an aerodynamic diameter ≤ 10 μm (PM_10_) exposure with bone health (4, 9). For each 1 μg/m^3^ increase in PM_2.5_, the prevalence of osteoporosis increased by 5% in all participants; per 1 μg/m^3^ increase in PM_10_ corresponded with a 4% elevation in the risks of osteoporosis in the rural population (15). These inconsistent results may contribute to heterogeneity in the basic characteristics of subjects, study design, sample size, measurement of outcomes, and covariate correction during the analysis of various studies. Similarly, inconsistent findings exist for the relationship between air pollution exposure and fractures. A cross-sectional study of 44,602 Korean women aged 50 years or older showed a positive association between PM_2.5_ exposure and osteoporotic fractures (7), while another cross-sectional study reported no significant association between PM exposure and forearm fractures in older adults (14).

Previous studies have typically focused on the association of a single air pollutant with disease risk, while largely ignoring the combined effects of various air pollutants (16, 17). It remains unclear whether combined exposure to various air pollutants could alter the associations between genetic factors and OP. Furthermore, previous studies, limited by cross-sectional studies and small sample designs, have only demonstrated associations and lacked cohort studies to confirm the relationship between air pollution and OP. More importantly, it is still unknown how air pollutants interact with genetic factors in determining the risk of OP. Therefore, it is necessary to conduct a large-scale cohort study to reveal the underlying relationship. Therefore, based on a large-scale cohort (UK Biobank) we conducted a systemic study to test associations between OP risk and exposure to environmental air pollutants (PM_2.5_, PM_10_, PM with diameters between 2.5 and 10 μm: PM_2.5−10_, nitrogen dioxide: NO_2_ and nitrogen oxide: NO_x_) in either single or multiple patterns, and also to test the joint effects of air pollution and genetic factors on the risk of OP.

## 2. Materials and methods

### 2.1. Study design

**Figure 1:**
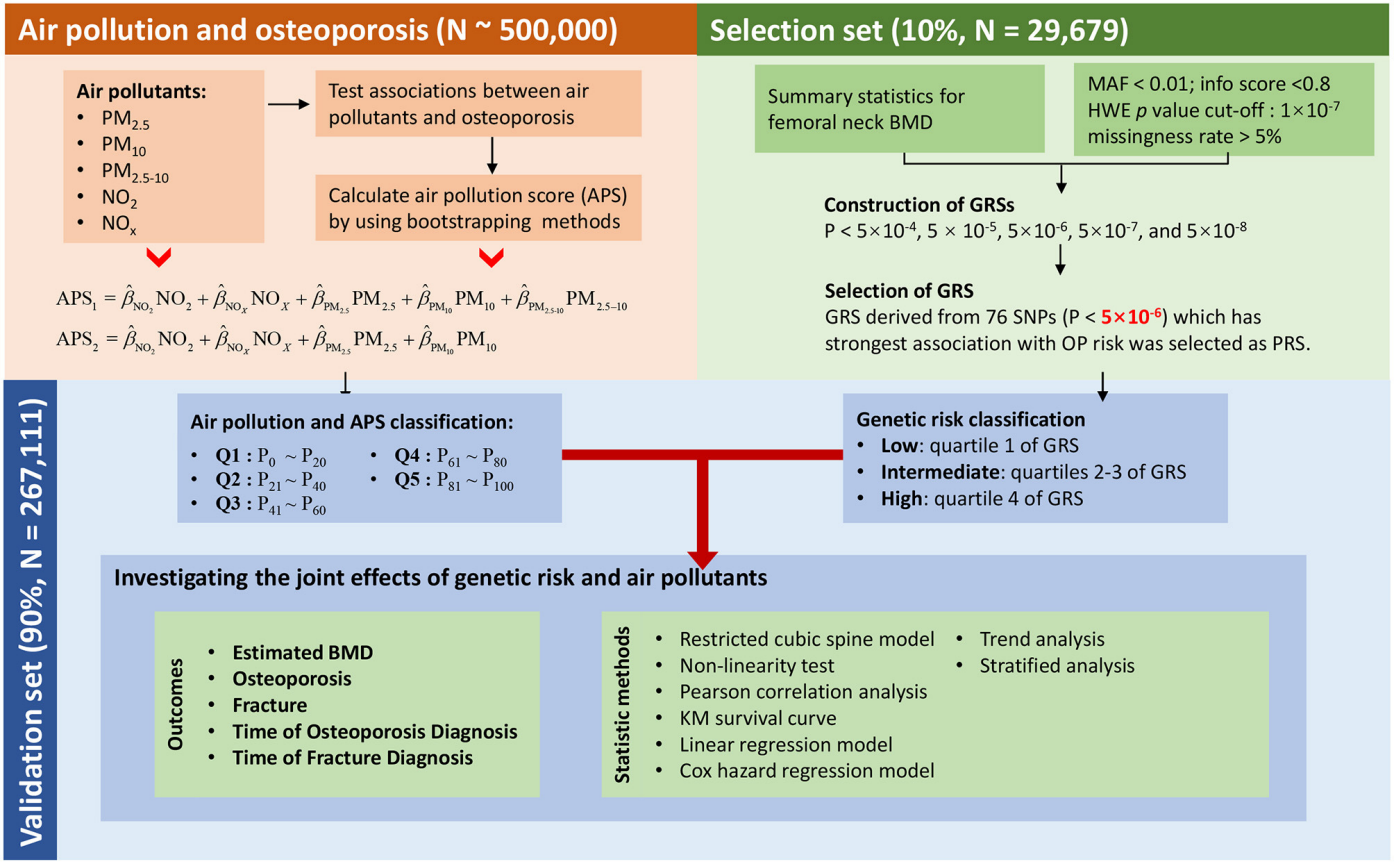
Study design and workflow.

For observational analysis, all individuals were used to assess the separate and joint effect of five types of air pollution on OP. For genetic analysis, around 296,790 European independent individuals were divided into a selection set (*N* = 29,679, 10%) and a validation set (*N* = 267,111, 90%). GRSs in different cut-offs were compared and best-performed GRS was finally selected in the selection set. All the other analyses were conducted in the validation set to avoid over-fitting. The overflow of our research was presented in Figure 1.

### 2.2. Data source

Between 2006 and 2010 the UK Biobanking Project recruited around 500,000 UK adults aged between 40 and 69 years, where ethical approval was certified by the Northwest Centre Research Ethics Committee, and informed consent was obtained from all participants. In our analysis, we included age (Field ID: 21022), gender (Field ID: 31), height (Field ID: 50), weight (Field ID: 21002), race (Field ID: 21000), eBMD (Field ID: 3084, 3148, 4105), eBMD T-score (Field ID: 77, 78, 4106), Fracture (Field ID: 6151), smoking status (Field ID: 20116), alcohol (Field ID: 20117), diet (Field ID: 100052), Townsend deprivation index (TDI) (Field ID: 189), International Classification of Diseases, Tenth Revision (ICD10).

### 2.3. Air pollution measurement

The researchers used the Land Use Regression (LUR) models, developed by the European Study of Cohorts for Air Pollution Effects (ESCAPE) project, to calculate the estimated annual average concentrations of ambient air pollution (PM_2.5_, PM_2.5−10_, PM_10_, NO_2_, and NO_x_) in UK Biobank (18, 19). Specifically, LUR models were used to assess variations of air pollutants concentrations at the residential address provided by the participants at baseline, and to estimate the individual exposure of the participants. Geographic Information System (GIS) variables were used as predictors (such as traffic intensity, population, topography, and land use) and a cross-validation procedure was used to assess model performance. Detailed processes for establishing LUR models for PM_2.5_, PM_2.5−10_, PM_10_, NO_2_, and NO_x_ have been described elsewhere (18, 19). In final, PM_2.5_, PM_10_, NO_2_ and NO_x_ had good model performance (cross-validation *R*^2^ = 77, 88, 87 and 88%, respectively), while PM_2.5−10_ had a relatively moderate model performance (cross-validation *R*^2^ = 57%). Exposure data for PM_2.5_, PM_2.5−10_, and NO_x_ were collected in 2010, while annual concentration data for NO_2_ and PM_10_ were within several years (NO_2_ in 2005, 2006, 2007, and 2010; PM_10_ in 2007 and 2010). The average values of NO_2_ and PM_10_ were calculated for further analysis. In addition, economic and cultural differences may lead to different exposures to air pollutants, so we included the TDI as a covariate in the subsequent analysis.

### 2.4. Measurements of OP

We considered three phenotypes for OP in our analysis, i.e., estimated bone mineral density (eBMD) (Field ID: 3084, 3148, 4105), the occurrence of OP, and fracture. Due to the lack of DXA (Dual Energy X-ray Absorptiometry)-BMD measurements in most participants in UK Biobank cohorts, we used eBMD which was measured for every subject as a measure of BMD, eBMD is BMD estimated by quantitative ultrasound of the heel (20). OP and fracture patients were defined by using ICD10 and the codes used in our analysis were presented as follows: OP (i.e., M80, M81, M82), fracture (i.e., M484, M485, M80, M843, M844, S12, S22, S32, S42, S52, S72, S82, Z8731, Z87310, Z87311). We excluded patients who were diagnosed before the baseline questionnaire was administered. We excluded individuals with unusual large or small eBMD value (eBMD < mean - 4.5SD (standard deviation) or eBMD > mean + 4.5SD). Furthermore, we applied strict quality control by using the following exclusion thresholds: SOS (speed of sound) (Male: [ ≤ 1,450 and ≥1,750 m/s], Female [ ≤ 1,455 and ≥1,700 m/s]) and BUA (broadband ultrasound attenuation) (Male: [ ≤ 27 and ≥138 dB/MHz], Female [ ≤ 22 and ≥138 dB/MHz]) for male and female subjects separately (20).

### 2.5. Calculation of APSs

According to previous research, we also created a weighted APS for OP by summing the weights of different air pollutants (17). Weights and corresponding confidence interval (CI) for different air pollutions are derived from the median and 2.5%, 75% of the bootstrapping distribution (21). In this procedure, we resampled the individuals with replacement, using the regenerated samples to assess the association between each air pollution and OP risk, with 1000 replications. Then the weighted APS was calculated through the combination of five air pollutants, weighted by the estimated coefficients on OP risk. Additionally, considering the weak association between PM_2.5−10_ and OP risk, we further constructed APS_2_ as a sensitivity analysis. The formulas of APS_1_ and APS_2_ were as follows:


(1) $$\begin{array}{l} {APS}_{1}={\hat{\beta }}_{{NO}_{2}}{NO}_{2}+{\hat{\beta }}_{{NO}_{x}}{NO}_{x}+{\hat{\beta }}_{{PM}_{2.5}}{PM}_{2.5}+{\hat{\beta }}_{{PM}_{10}}{PM}_{10}\\ \;+{\hat{\beta }}_{{PM}_{2.5-10}}{PM}_{2.5-10} \end{array}$$

(2) $$\begin{array}{lll} {APS}_{2} & = & {\hat{\beta }}_{{NO}_{2}}{NO}_{2}+{\hat{\beta }}_{{NO}_{x}}{NO}_{x}+{\hat{\beta }}_{{PM}_{2.5}}{PM}_{2.5}+{\hat{\beta }}_{{PM}_{10}}{PM}_{10} \end{array}$$

### 2.6. Construction of GRS

To ensure quality, strict quality controls for induvial were conducted in genetic analysis: (1) failed genotyping samples were removed; (2) non-white British ancestry samples were removed; (3) samples without principal components calculations were removed; (4) genetically correlated individuals were removed; (5) individuals with sex chromosome aneuploid were removed. Finally, a total of 335,198 white British individuals were reserved for further analysis. Considering that the BMD of the femoral neck (FN-BMD) is the gold standard for diagnosing OP, we downloaded summary statistics for the largest GWAS of FN-BMD conducted by GEnetic Factors for OP Consortium (GEFOS, http://www.gefos.org), which is publicly available (22). The genotype of each SNP was obtained from the UK Biobank and the quality control of SNPs was described elsewhere (23). A total of ~92 million variants were generated by imputation based on Haplotype Reference Consortium (HRC), 10 thousand individuals from the UK and 1000 Genomes reference panels. We removed low quality SNPs by following criteria: (1) minor allele frequency (MAF) < 0.01; (2) info score < 0.8; (3) Hardy-Weinberg equilibrium (HWE) *p-*value cut-off was set to 1 × 10^−7^; (4) and missingness rate > 5%. Finally, ~9,400,000 SNPs remained for further analysis. Lead SNPs for FN-BMD were determined by using the “–clump” procedure in PLINK software (24) at different thresholds (*p* < 5 × 10^−4^, 5 × 10^−5^, 5 × 10^−6^, 5 × 10^−7^, 5 × 10^−8^), containing 1498, 252, 76, 38 and 24 SNPs, respectively. The equation could be expressed as: $\text{GR}{\text{S}}_{i}\;=\;\overset{K}{\underset{k\;=\;1}{\sum }}G_{ik}{\hat{\theta }}_{k}$, where ${\hat{\theta }}_{k}$ is the SNP estimated effect for the *k*^th^ SNP, and *G*_*ik*_ is the genotypes (0, 1, 2) of the *k*^th^ SNP on *i*^th^ individual. Finally, we compared these five GRSs generated in different thresholds in the selection set after adjusting for age, sex, TDI, genotyping chip (UKB vs. BiLEVE), and top 10 genetic principal components (PCs). Finally, best-performed GRS (*p* < 5 × 10^−6^) with the highest effect on OP was chosen to represent genetic components (Supplementary Table S2). And the SNPs identified at the significance of 5 × 10^−6^ used for the construction of GRS were presented in Supplementary Table S3.

### 2.7. Statistical analysis

Pearson correlation was used to assess the relationship between air pollutants, and linear regression models were used to assess the associations between air pollutants and eBMD. A multivariable cox regression model was used to evaluate the association and hazard ratios (HR) were calculated with 95% CI after adjusting for several covariates. The restricted cubic spline (RCS) method was used to evaluate non-linear relationships between air pollutants and OP risk. Trend analysis was performed by Cochran-Armitage trend test with the “DescTools” package (25). It was noted that, for observational analysis, age, gender, genotyped batch, TDI, height, weight, and smoking status were used as covariates. For GRS analysis, age, gender, TDI, smoking status, genotyped batch, height, weight, and top 10 PCs were used as covariates. For sensitivity analysis, batch, center, age, sex, race, Townsend deprivation index, height, weight, smoking status, alcohol, physical activity, diet, CKD, T2D, cancer, and deprivation were used as covariates. To better evaluate the diseases risk among participants with different genetic risks, we divided GRS selected into different groups: low genetic risk (bottom quintile of GRS), intermediate genetic risk (quintiles 2 to 4), and high genetic risk (top quintile). All statistical analysis was performed in *R* 3.6.1 and the statistical significance was set to two-side *P* < 0.05.

## 3. Result

### 3.1. Basic information

In this study, a total of 13,291 OP cases and 19,695 fracture cases were recorded among 430,120 participants. The baseline characteristics of the study participants are presented in Table 1. Participants who had OP or fracture were older, predominantly female, had higher smoking rates, and with lighter weight and shorter height compared with those without OP or fracture. The mean of estimates of PM_2.5_, PM_10_, PM_2.5−10_, NO_2_ and NO_x_ were 9.98 μg/m^3^, 19.28 μg/m^3^, 6.42 μg/m^3^, 29.12 μg/m^3^ and 43.86 μg/m^3^.

**Table 1:** Baseline characteristics of the study subjects in the UK Biobank cohort.

**Variables**	**Levels**	**Overall**	**No OP**	**OP**	**No Fracture**	**Fracture**
		***N** =* **430,120**	***N** =* **416,829 (96.9%)**	***N** =* **13,291 (3.1%)**	***N** =* **410,425 (95.4%)**	***N** =* **19,695 (4.6%)**
Age [mean (SD)]		56.54 (8.09)	56.38 (8.09)	61.48 (6.13)	56.40 (8.09)	59.45 (7.46)
Sex (%)	female	233,356 (54.3)	222,217 (53.3)	11,139 (83.8)	220,401 (53.7)	12,955 (65.8)
	male	196,764 (45.7)	194,612 (46.7)	2,152 (16.2)	190,024 (46.3)	6,740 (34.2)
Height [mean (SD)]		168.54 (9.26)	168.71 (9.25)	163.13 (8.08)	168.62 (9.26)	166.96 (9.12)
Weight [mean (SD)]		78.06 (15.86)	78.32 (15.82)	69.87 (14.73)	78.15 (15.84)	76.17 (16.00)
TDI [mean (SD)]		−1.38 (3.02)	−1.39 (3.02)	−1.13 (3.15)	−1.39 (3.02)	−1.18 (3.12)
Race (%)	White	406,601 (94.5)	393,771 (94.5)	12,830 (96.5)	387,412 (94.4)	19,189 (97.4)
	Asian	8,484 (2.0)	8,279 (2.0)	205 (1.5)	8,273 (2.0)	211 (1.1)
	Black	6,809 (1.6)	6,723 (1.6)	86 (0.6)	6,718 (1.6)	91 (0.5)
	Mixed	8,226 (1.9)	8,056 (1.9)	170 (1.3)	8,022 (2.0)	204 (1.0)
Smoking status (%)	Never	235,616 (54.8)	228,677 (54.9)	6,939 (52.2)	225,503 (54.9)	10,113 (51.3)
	Previous	150,216 (34.9)	145,367 (34.9)	4,849 (36.5)	142,940 (34.8)	7,276 (36.9)
	Current	44,288 (10.3)	42,785 (10.3)	1,503 (11.3)	41,982 (10.2)	2,306 (11.7)
eBMD [mean (SD)]		0.55 (0.14)	0.55 (0.14)	0.45 (0.12)	0.55 (0.14)	0.49 (0.13)
No_x_ [mean (SD)]		43.86 (15.59)	43.84 (15.58)	44.55 (15.94)	43.84 (15.57)	44.36 (16.01)
NO_2_ [mean (SD)]		29.12 (9.19)	29.11 (9.18)	29.53 (9.33)	29.11 (9.18)	29.33 (9.27)
PM_10_ [mean (SD)]		19.28 (1.95)	19.28 (1.95)	19.32 (1.93)	19.28 (1.95)	19.27 (1.94)
PM_2.5_ [mean (SD)]		9.98 (1.06)	9.98 (1.06)	10.05 (1.07)	9.98 (1.06)	10.02 (1.08)
PM_2.5−10_ [mean (SD)]		6.42 (0.90)	6.42 (0.90)	6.43 (0.89)	6.42 (0.90)	6.42 (0.89)

OP, osteoporosis; NO_x_, nitrogen oxides; NO_2_, nitrogen dioxide; PM_2.5_, particulate matter with an aerodynamic diameter ≤ 2.5 μm; PM_10_, particulate matter with an aerodynamic diameter ≤ 10 μm; PM_2.5−10_, particulate matter with an aerodynamic diameter between 2.5 and 10 μm; eBMD, estimated bone mineral density; TDI, Townsend deprivation index; SD, standard deviation.

### 3.2. Relationship between air pollution and OP risk

**Figure 2:**
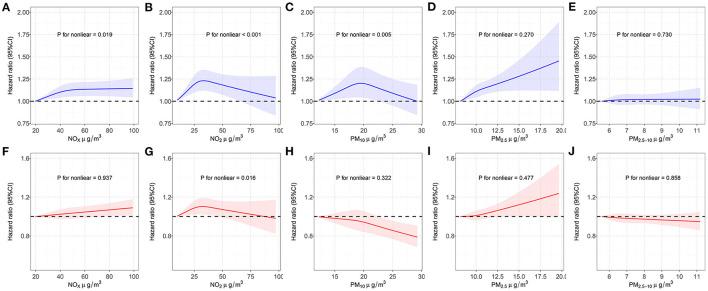
Restricted cubic spline models for the relationship between air pollution and OP and fracture risk. **(A)** NO_x_ concentration and OP risk; **(B)** NO_2_ concentration and OP risk; **(C)** PM_10_ concentration and OP risk; **(D)** PM_2.5_ concentration and OP risk; **(E)** PM_2.5−10_ concentration and OP risk; **(F)** NO_x_ concentration and fracture risk; **(G)** NO_2_ concentration and fracture risk; **(H)** PM_10_ concentration and fracture risk; **(I)** PM_2.5_ concentration and fracture risk; **(J)** PM_2.5−10_ concentration and fracture risk. Associations were adjusted for age, sex, genotyped batch, Townsend deprivation index, height, weight, and smoking status. OP, osteoporosis; NO_x_, nitrogen oxides; NO_2_, nitrogen dioxide; PM_2.5_, particulate matter with an aerodynamic diameter ≤2.5 μm; PM_10_, particulate matter with an aerodynamic diameter ≤10 m; PM_2.5−10_, particulate matter with an aerodynamic diameter between 2.5 and 10 μm; CI, confidence interval.

Five air pollutants were involved in our analyses: PM_2.5_, PM_10_, PM_2.5−10_, NO_2_ and NO_x_. The concentration of PM_2.5_, PM_10_, NO_2_ and NO_x_ were significantly correlated with the decrease of eBMD even after Bonferroni adjustment (Supplementary Table S4). Similar results were obtained from sensitivity analysis (Supplementary Table S5). Then we used RCS model to assess the associations between each air pollutant with OP and fracture risk (Figure 2). As shown in Supplementary Table S6, PM_2.5_, NO_2_ and NO_x_ were significantly associated with an increased risk of OP. The HR of OP occurring in subjects were estimated to be 1.046 (95% CI = 1.027–1.066, *P* = 2.31 × 10^−6^), 1.029 (95% CI = 1.008–1.049, *P* = 0.005) and 1.029 (95% CI = 1.011–1.048, *P* = 0.002) for PM_2.5_, NO_2_ and NO_x_, respectively. However, we did not observe a significant association between PM_10_ (HR = 1.009, 95% CI, 0.990–1.028, *P* = 0.357) or PM_2.5−10_ (HR = 1.007, 95% CI, 0.990–1.024, *P* = 0.431) and risk of OP. Sensitivity analysis did not have a significant impact on the results (Supplementary Table S7). RCS method was then used to explore the linear relationships between each air pollutant and OP risk, we observed non-linear relationships for PM_10_, NO_2_, NO_x_ and a linear relationship for PM_2.5_, PM_2.5−10_. Supplementary Table S8 shows the associations between individual air pollutants and fracture. PM_2.5_ (HR = 1.018, 95% CI = 1.003–1.034, *P* = 0.020), NO_2_ (HR = 1.022, 95% CI = 1.005–1.039, *P* = 0.009), and NO_x_ (HR = 1.023, 95% CI = 1.007–1.038, *P* = 0.003) concentrations were interrelated with an increased risk of fracture. In addition, we observed a non-significant effect on risk of fracture with the increases in PM_10_ (HR = 0.987, 95% CI = 0.971–1.002, *P* = 0.087) and PM_2.5−10_ (HR = 0.994, 95% CI = 0.980–1.009, *P* = 0.443). Sensitivity analysis showed that PM_2.5_ no longer increased the risk of fracture (HR = 1.009, 95% CI, 0.993–1.025, *P* = 0.275) (Supplementary Table S9). For fracture risk, we observed a non-linear relationship for NO_2_ and linear relationships for PM_2.5_, PM_10_, PM_2.5−10_, NO_x_ by using RCS model. Then the stratified analyses were also conducted, detailed results were presented in Supplementary Tables S10, S11.

### 3.3. Relationship between APSs and OP risk

**Figure 3:**
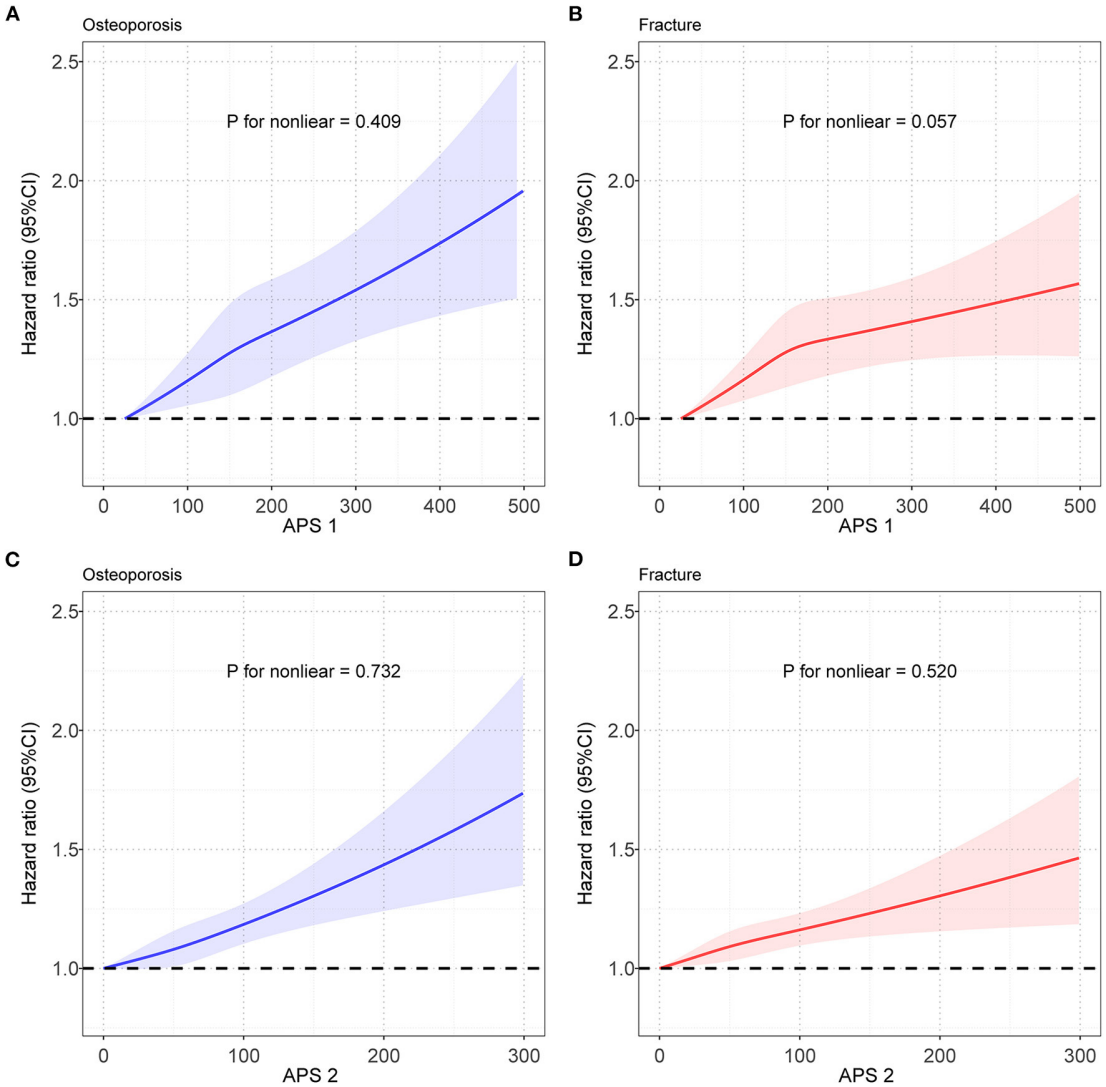
Restricted cubic spline models for the relationship between APS and OP and fracture risk. **(A)** Relationship between APS_1_ and OP risk; **(B)** Relationship between APS_1_ and fracture risk; **(C)** Relationship between APS_2_ and OP risk; **(D)** Relationship between APS_2_ and fracture risk. Note: Associations were adjusted for age, sex, genotyped batch, Townsend deprivation index, height, weight, and smoking status. OP, osteoporosis; APS, air pollution score; CI, confidence interval.

We constructed APSs by combining different air pollutants with the bootstrapping procedure, and correlations between APSs and five air pollutants were shown in Supplementary Figure S1. The RCS model was used to evaluate the relationship between APS and the risk of OP and fracture. As shown in Figure 3, the spline analysis showed a significant relationship between APS and the risk of OP and fracture (*P* for non-linear was 0.409 and 0.057, respectively, in the OP and fracture group). Similar results between APS without PM_2.5−10_ and the risk of OP and fracture (*P* for nonlinear was 0.732 and 0.520, respectively, in the OP and fracture group) were observed (Figure 3). The relationships between APS and OP and fracture were shown in Supplementary Table S12. We found that higher levels of APSs were associated with a higher risk of developing OP. From the first quintile (Q1) of APS to the fifth quintile (Q5) of APS, we found an incremental trend in OP risk. The HRs (95% CI) of OP occurring in subjects with higher quintile groups compared to those with the lowest quintile of the APS were estimated to be 1.027 (0.967–1.090), 1.049 (0.988–1.115), 1.094 (1.030–1.161), and 1.140 (1.072–1.213). After excluding PM_2.5−10_ in the APS, the result did not change appreciably. The HRs (95% CI) of OP were estimated to be 1.082 (1.019–1.149), 1.016 (0.956–1.080), 1.121 (1.056–1.190), and 1.173 (1.103–1.247) in higher quintile groups, when compared with Q1 of the APS. Similar results were observed in the relationship between APS and fracture. APSs were associated with an increased risk of fracture in a dose-response relationship. Compared with the lowest quintile of the APS, the HRs (95% CI) of fracture were estimated to be 1.005 (0.956–1.055), 1.040 (0.990–1.092), 1.064 (1.013–1.117), and 1.080 (1.026–1.136) for the higher quintile groups. Similarly, the removal of PM_2.5−10_ in the APS did not significantly affect the results. The HRs (95% CI) of fracture for those exposed to the higher quintile groups were estimated to be 1.004 (0.956–1.055), 1.042 (0.992–1.094), 1.071 (1.020–1.125), and 1.077 (1.024–1.132), respectively. Sensitivity analysis did not have a significant impact on the results (Supplementary Table S13).

### 3.4. The joint effect of air pollution and genetic risk scores on OP risk

To further explore the joint effect of genetic and environmental factors on OP, we constructed a BMD-based GRS (detailed information was provided in Methods). The histograms show that the GRS of BMD is normally distributed and well stratified in osteoporosis/fracture patients and controls (Supplementary Figures S2A, B). And the RCS curves showed a significant negative linear relationship between GRS and osteoporosis/ fracture risk, respectively (Supplementary Figures S2C, D). Therefore, we believed that GRS could well represent the genetic component of the osteoporosis phenotype.

As the concentration of different air pollutant rises, the risk of OP in participants who has the intermediate GRS and low GRS increases significantly. We also found that participants with low GRS and the highest air pollutant concentration had the highest risk of OP, the HRs (95% CI) of PM_2.5_, PM_10_, PM_2.5−10_, NO_2_, and NO_x_ on OP risk were estimated to be 1.706 (1.483–1.964), 1.658 (1.434–1.916), 1.696 (1.478–1.947), 1.740 (1.506–2.001) and 1.659 (1.442–1.908), respectively. In addition, the risk of OP was higher in the low GRS group than in the intermediate GRS group at equal concentrations of single air pollutants, while no such results were observed in the high GRS group. Similar results were also observed in the fracture individuals (Supplementary Figure S3).

**Figure 4:**
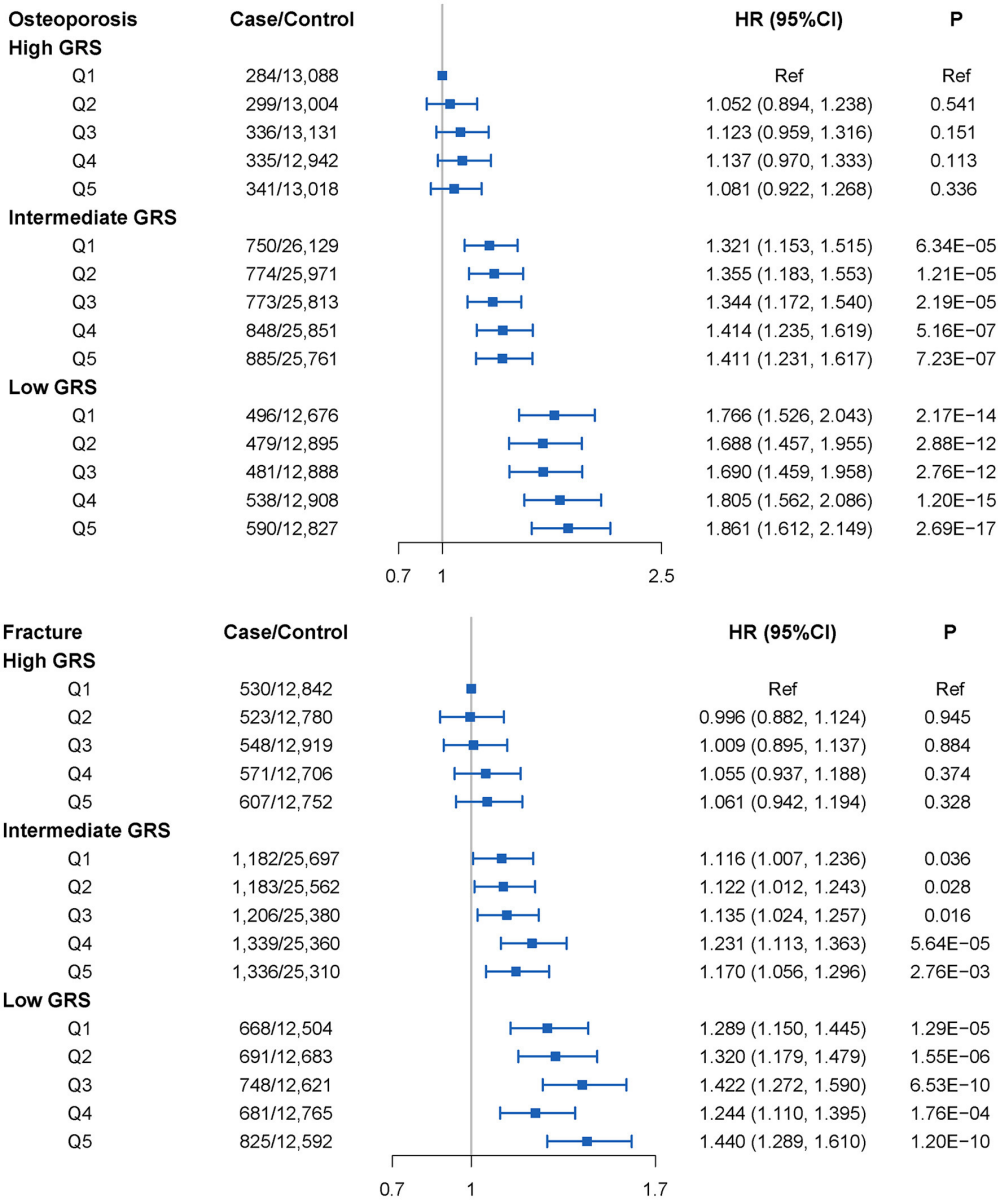
OP and fracture risks in the subgroups stratified by genetic risk and air pollution scores (APS_1_) concentrations (vs. participants with the lowest concentration of APS_1_ in the highest genetic risk group) in the UKB cohort. Note: Associations were adjusted for age, sex, genotyped batch, Townsend deprivation index, height, weight, smoking status, and the first 10 principal components of ancestry. CI, confidence interval; HR, hazard ratio; GRS, genetic risk score.

We next assessed the joint association of APS and GRS on the risk of developing OP. As shown in Figure 4, the joint effect of intermediate GRS or low GRS and APS increased the risk of developing OP, and from Q1 to Q5, we also found an approximate gradient increase in OP risk. However, these were not observed in the high GRS group. We also found that participants with high APS and low GRS had the highest risk of developing OP, 86.1% (95% CI = 61.2–114.9%) greater than participants with low APS and high GRS. Similar results were observed in the joint effect of GRS and APS on fracture. Participants with high APS and low GRS had a 44.0% (95% CI = 28.9–61.0%) higher fracture risk than those with low APS and high GRS. Similar results were obtained from sensitivity analysis (Supplementary Table S14). After excluding PM_2.5−10_ in the APS, the result did not change appreciably (Supplementary Figure S4 and Supplementary Table S15).

## 4. Discussion

This study observed the significant associations between an increased OP and fracture risk and the exposure to various ambient air pollutants, including PM_2.5_, PM_10_, PM_2.5−10_, NO_2_, and NO_x_. We then constructed APSs to evaluate the combined effect of various ambient air pollutants and found significant associations with the risk of developing OP and fracture. Moreover, we investigated the joint effects of genetic risk and air pollutants and found that low genetic risk and high APSs synergistically increased the risk of developing OP and fractures. In conclusion, this study performed a systemic study to disclose the associations between air pollution exposure and OP risk and highlighted the combined effects of multiple air pollutants and their interaction effects with genetic factors on OP risk.

In this large-scale prospective study, PM_2.5_ was significantly associated with the risk of OP and fracture, which is consistent with previous epidemiology studies. An OP sub-study of the population-based Oslo Health Study showed that total body BMD was negatively associated with both PM_2.5_ (3). Two studies in China and Italy found significant associations between OP and PM_2.5_ (9, 11). Furthermore, a study conducted in Korea showed that PM_2.5_ was associated with fracture (7). The association between PM_10_ and OP and fracture is inconsistent in previous studies (6, 7, 11), while our study identified adverse effects of PM_10_ on bone metabolism. In our analysis, we detected a significant effect of raised PM_10_ concentration on eBMD, but no significant association with OP and fracture risk. Our study found a nonlinear relationship between PM_10_ and OP risk using the RCS method, which may be one of the reasons for the inconsistent findings between BMD and OP. Additionally, the inconsistent results between PM_2.5_ and PM_10_ may be because PM_2.5_ has a larger specific surface area compared to PM_10_ and can adsorb more compounds and metals (26), which could affect the balance of bone metabolism. We also confirmed that NO_2_ and NO_x_ were significant air pollutants related to the risk of OP and fracture, which was consistent with previous studies. Mazzucchelli et al. (5) found that hip fracture incidence was associated with SO_2_, NO, and NO_2_ (5). Retrospective cohort studies in Asia showed that NO_2_ was associated with an increased risk of OP (4, 9). In addition, NO, NO_2_ and NO_x_ were found negatively associated with BMD T-scores in a cross-sectional study (6).

The importance of assessing exposure to multiple ambient air pollutants has been recognized in recent years. Air pollution is a complex mixture composed of many substances (27), and synergistic effects may exist among various air pollutants. It is difficult to sort out the effects of a single component on humans. Therefore, APS enables a better assessment of combined exposure to air pollutants. As we expected, this study found more stable and robust associations between APS and the risk of OP and fracture, when compared to single pollutants. Similar methods have been used in previous studies. Lin et al. (6) found that the joint effects of SO_2_ and NO_2_, CO, and NO_x_ on OP were more significant than individual air pollutants (6). Moreover, similar methods have been used to evaluate the joint effects of other environmental risks (28, 29) and dietary factors (30).

The biological mechanisms underlying the effects of ambient air pollutants on OP risk have not been clearly explained, however, previous studies have presented several possible mechanisms for the relationship between air pollutants and OP. Air pollutants could lead to oxidative stress and inflammation (31, 32). Oxidative stress causes DNA damage and cellular aging which disrupts the balance between bone resorption and osteogenesis (33, 34). It has been demonstrated that exposure to air pollutants such as NO_2_ and PM can lead to the production of free radicals, and then cause inflammatory processes (35–39). Pro-inflammatory cytokines such as tumor necrosis factor-α (TNF-α), interleukin (IL)-1β, IL-6, and IL-17 can affect the differentiation and function of osteoblasts and osteoclasts during bone metabolism (40–42), thus bring out the imbalance in the bone homeostasis and decrease bone density. For example, TNF-α drives RANK expression in monocytes and stimulates monocytes' conversion into osteoclast precursors, in addition to promoting RANKL expression in stromal cells (43). Air pollutants can also induce immune responses. Chronic exposure to PM has significant effects on innate and adaptive immune cell populations in the lung, lymphatic, and systemic immune populations have been previously reported. Chemicals attached to PM such as polycyclic aromatic hydrocarbons (PAH) can enhance T helper (Th)17 lymphocyte differentiation (44). Air pollutants can affect vitamin D synthesis by increasing levels of parathyroid hormone (45, 46), this hormone also facilitates the differentiation of T cells into Th17 cells (47). IL-17 is secreted by Th17 and can induce osteoblast production by promoting the release of RANKL from osteoblasts and osteocytes, and has an important role in bone metabolism. IL-17 also enhances RANKL sensitivity by regulating RANK expression, leading to increased osteoclast numbers and bone resorption (48). In addition, some indirect factors, such as vitamin D deficiency, can also link air pollution and OP. Prolonged exposure to high air pollution levels increases the risk of vitamin D deficiency (49). For example, benzo [a] pyrene carried by PM_2.5_ could promote the catabolism of vitamin D_3_ (50). Environmental air pollutants such as PM and ozone can block ultraviolet light from the earth's surface (51), and that severe air pollution may also reduce the frequency of outdoor activities, which is detrimental to vitamin D synthesis.

We hypothesize that multiple air pollutants may influence OP risk through similar biological mechanisms such as oxidative stress and inflammation. In addition, studies have demonstrated interactions and synergistic effects between CO and NO_x_, as well as SO_2_ and NO_2_ on BMD, which then reduces the efficiency of O_2_ transport and the reversible (NO) or irreversible (CO) inhibition of mitochondrial oxidative phosphorylation by binding to hemoglobin. or irreversible (CO) inhibition of mitochondrial oxidative phosphorylation by reversible binding to the heme aa3 site of cytochrome c oxidase (52). Therefore, we hypothesized that NO_x_ exacerbates CO-induced hypoxia and exacerbates OP risk. NO_2_ can promote sulfate formation, which, due to hygroscopicity, can form aqueous layers on mineral oxide particles, leading to further adsorption of and reaction with other pollutants, including SO_2_ (53). Therefore, we hypothesize that the synergistic effect of SO_2_ and NO_2_ may be a risk factor for OP by promoting the adsorption of other pollutants.

The novelty of this study is the prospective design and the large sample size. The present study is based on a large-scale UK Biobank cohort including approximately 500,000 participates and therefore has good statistical power. In addition, we assessed the role of air pollutants in the association between genetic factors and OP, allowing us to accurately determine the effects of air pollutants on populations with different susceptibility levels. Furthermore, cross-validation analyses were performed in this study and the air pollutant models were found to perform well, demonstrating the robustness of our findings. However, there also exist some limitations: First, due to the big cost of performing DXA-BMD measurements in cohorts with a large sample size, eBMD instead of BMD was used as an indicator of bone strength. Although the previous study has shown the high consistency between genetic determined using ultrasound-derived BMD measurements and those using DXA-derived BMD, some significant differences still exist (54). Second, although we have comprehensively considered a variety of air pollutants, some previously reported air pollutants such as O_3_, SO_2_, and CO are not present in the UK Biobank. Third, we constructed the APS by treating air pollutants as linear indicators, and although consistent results were shown across phenotypes, possible non-linear relationships between individual air pollutants and OP could interfere with the true association. Finally, the participants in the UK Biobank are predominantly of European origin. The applicability of the findings obtained from this study to other ethnic groups and regions requires further investigation.

## 5. Conclusion

In conclusion, we found that chronic exposure to air pollution, assessed with APS, played an important role in improving the risk of developing OP and fractures, and increased the adverse effects of genetic risk. Our findings emphasize that improving air quality can reduce the risk of developing OP and fracture, which has important implications for the development of environmental health policies.

## Data availability statement

The original contributions presented in the study are included in the article/Supplementary material, further inquiries can be directed to the corresponding authors.

## Ethics statement

Written informed consent was obtained from the individual(s) for the publication of any potentially identifiable images or data included in this article.

## Author contributions

F-YD, S-FL, and X-HY conceived the design of the study. X-HY and LB obtained the data. X-HY cleared up the datasets. X-HY and H-WC mainly performed the data analyses. F-YD, S-FL, X-HY, and H-WC drafted and revised the manuscript. All authors approved the manuscript and provided relevant suggestions.

## References

[1] Hendrickx G Boudin E Van Hul WA. Look behind the scenes: the risk and pathogenesis of primary osteoporosis. Nat Rev Rheumatol. (2015) 11:462–74. 10.1038/nrrheum.2015.48 25900210

[2] Pinault. Estimates and 25-year trends of the global burden of disease attributable to ambient air pollution: an analysis of data from the Global Burden of Diseases Study 2015. Lancet. (2017) 389:E15. 10.1016/S0140-6736(17)30505-6 28408086 PMC5439030

[3] Alvaer K Meyer HE Falch JA Nafstad P Sogaard AJ. Outdoor air pollution and bone mineral density in elderly men - the Oslo Health Study. Osteoporos Int. (2007) 18:1669–74. 10.1007/s00198-007-0424-y 17619807

[4] Chang KH Chang MY Muo CH Wu TN Hwang BF Chen CY. Exposure to air pollution increases the risk of osteoporosis: a nationwide longitudinal study. Medicine. (2015) 94:e733. 10.1097/MD.0000000000000733 25929905 PMC4603067

[5] Mazzucchelli R Crespi Villarias N Perez Fernandez E Durban Reguera ML Garcia-Vadillo A Quiros FJ. Miguel A. Short-term association between outdoor air pollution and osteoporotic hip fracture *Osteoporos Int*. (2018) 29:2231–41. 10.1007/s00198-018-4605-7 30094608

[6] Lin YH Wang CF Chiu H Lai BC Tu HP Wu PY. Air pollutants interaction and gender difference on bone mineral density T-Score in Taiwanese adults. Int J Environ Res Public Health. (2020) 17:24. 10.3390/ijerph17249165 33302461 PMC7764089

[7] Sung JH Kim K Cho Y Choi S Chang J Kim SM. Association of air pollution with osteoporotic fracture risk among women over 50 years of age. J Bone Miner Metab. (2020) 38:839–47. 10.1007/s00774-020-01117-x 32507945

[8] Ranzani OT Mila C Kulkarni B Kinra S Tonne C. Association of ambient and household air pollution with bone mineral content among adults in Peri-urban South India. JAMA Netw Open. (2020) 3:e1918504. 10.1001/jamanetworkopen.2019.18504 31899531 PMC6991311

[9] Qiao D Pan J Chen G Xiang H Tu R Zhang X. Long-term exposure to air pollution might increase prevalence of osteoporosis in Chinese rural population. Environ Res. (2020) 183:109264. 10.1016/j.envres.2020.109264 32311909

[10] Shin J Kweon HJ Kwon KJ Han SH. Incidence of osteoporosis and ambient air pollution in South Korea: a population-based retrospective cohort study. BMC Public Health. (2021) 21:1794. 10.1186/s12889-021-11866-7 34610796 PMC8493748

[11] Adami G Cattani G Rossini M Viapiana O Olivi P Orsolini G. Association between exposure to fine particulate matter and osteoporosis: a population-based cohort study. Osteoporos Int. (2022) 33:169–76. 10.1007/s00198-021-06060-9 34268604 PMC8758604

[12] Wu J Guo B Guan H Mi F Xu J Basang LY. The association between long-term exposure to ambient air pollution and bone strength in China. J Clin Endocrinol Metab. (2021) 106:e5097–108. 10.1210/clinem/dgab462 34263315

[13] Yang Y Li R Cai M Wang X Li H Wu Y. Ambient air pollution, bone mineral density and osteoporosis: results from a national population-based cohort study. Chemosphere. (2023) 310:136871. 10.1016/j.chemosphere.2022.136871 36244420

[14] Alver K Meyer HE Falch JA Søgaard AJ. Outdoor air pollution, bone density and self-reported forearm fracture: the Oslo Health Study. Osteoporos Int. (2010) 21:1751–60. 10.1007/s00198-009-1130-8 20077108

[15] Zhang F Zhou F Liu H Zhang X Zhu S Zhang X. Long-term exposure to air pollution might decrease bone mineral density T-score and increase the prevalence of osteoporosis in Hubei province: evidence from China Osteoporosis Prevalence Study. Osteoporos Int. (2022) 33:2357–68. 10.1007/s00198-022-06488-7 35831465

[16] Pang KL Ekeuku SO Chin KY. Particulate air pollution and osteoporosis: a systematic review. Risk Manag Healthc Policy. (2021) 14:2715–32. 10.2147/RMHP.S316429 34194253 PMC8238075

[17] Wang M Zhou T Song Y Li X Ma H Hu Y. exposure to various ambient air pollutants and incident heart failure: a prospective analysis in UK Biobank. Eur Heart J. (2021) 42:1582–91. 10.1093/eurheartj/ehaa1031 33527989 PMC8060055

[18] Eeftens M Beelen R de Hoogh K Bellander T Cesaroni G Cirach M. Development of land use regression models for PM(2.5), PM(2.5) absorbance, PM(10) and PM(coarse) in 20 European study areas; results of the ESCAPE project. Environ Sci Technol. (2012) 46:11195–205. 10.1021/es301948k 22963366

[19] Beelen R Hoek G Vienneau D Eeftens M Dimakopoulou K. Development of NO_2_ and NOx land use regression models for estimating air pollution exposure in 36 study areas in Europe – The ESCAPE project. Atmos Environ. (2013) 72:10–23. 10.1016/j.atmosenv.2013.02.037

[20] Morris JA Kemp JP Youlten SE Laurent L Logan JG Chai RC. An atlas of genetic influences on osteoporosis in humans and mice. Nat Genet. (2019) 51:258–66. 10.1038/s41588-018-0302-x 30598549 PMC6358485

[21] Efron B Tibshirani RJ. An Introduction to the Bootstrap. London: CRC Press. (1994).

[22] Zheng HF Forgetta V Hsu YH Estrada K. Rosello-Diez A, Leo PJ, Dahia CL, Park-Min KH, Tobias JH, Kooperberg C. Whole-genome sequencing identifies EN1 as a determinant of bone density and fracture. Nature. (2015) 526:112–7. 26367794 10.1038/nature14878 PMC4755714

[23] Yu XH Wei YY Zeng P Lei SF. Birth weight is positively associated with adult osteoporosis risk: observational and Mendelian randomization studies. J Bone Miner Res. (2021) 9:4316. 10.1002/jbmr.4316 34105796

[24] Purcell S Neale B Todd-Brown K Thomas L Ferreira MA Bender D. a tool set for whole-genome association and population-based linkage analyses. Am J Hum Genetics. (2007) 81:559–75. 10.1086/519795 17701901 PMC1950838

[25] Agresti A. Categorical Data Analysis. New York, NY: John Wiley and Sons. (2003).

[26] Zou Y Jin C Su Y Li J Zhu B. Water soluble and insoluble components of urban PM2.5 and their cytotoxic effects on epithelial cells (A549) in vitro. Environ Pollut. (2016) 212:627–35. 10.1016/j.envpol.2016.03.022 27039898

[27] Kurt OK Zhang J Pinkerton KE. Pulmonary health effects of air pollution. Curr Opin Pulm Med. (2016) 22:138–43. 10.1097/MCP.0000000000000248 26761628 PMC4776742

[28] Vassos E Sham P Kempton M Trotta A Stilo SA Gayer-Anderson C. The Maudsley environmental risk score for psychosis. Psychol Med. (2020) 50:2213–20. 10.1017/S0033291719002319 31535606 PMC7557157

[29] Padmanabhan JL Shah JL Tandon N Keshavan MS. The “polyenviromic risk score”: Aggregating environmental risk factors predicts conversion to psychosis in familial high-risk subjects. Schizophr Res. (2017) 181:17–22. 10.1016/j.schres.2016.10.014 28029515 PMC5365360

[30] Zhang Y Yang H Li S Li WD Wang Y. Consumption of coffee and tea and risk of developing stroke, dementia, and poststroke dementia: a cohort study in the UK Biobank. PLoS Med. (2021) 18:e1003830. 10.1371/journal.pmed.1003830 34784347 PMC8594796

[31] Araujo JA. Particulate air pollution, systemic oxidative stress, inflammation, and atherosclerosis. Air Qual Atmos Health. (2010) 4:79–93. 10.1007/s11869-010-0101-8 21461032 PMC3040314

[32] Lodovici M Bigagli E. Oxidative stress and air pollution exposure. J Toxicol. (2011) 2011:487074. 10.1155/2011/487074 21860622 PMC3155788

[33] Chandra A Rajawat J. Skeletal aging and osteoporosis: mechanisms and therapeutics. Int J Mol Sci. (2021) 22:553. 10.3390/ijms22073553 33805567 PMC8037620

[34] Almeida M Han L Martin-Millan M Plotkin LI Stewart SA Roberson PK. Skeletal involution by age-associated oxidative stress and its acceleration by loss of sex steroids. J Biol Chem. (2007) 282:27285–97. 10.1074/jbc.M702810200 17623659 PMC3119455

[35] Lorenzo J Horowitz M Choi Y. Osteoimmunology: interactions of the bone and immune system. Endocr Rev. (2008) 29:403–40. 10.1210/er.2007-0038 18451259 PMC2528852

[36] Moller P Loft S. Oxidative damage to DNA and lipids as biomarkers of exposure to air pollution. Environ Health Perspect. (2010) 118:1126–36. 10.1289/ehp.0901725 20423813 PMC2920082

[37] Yang W Omaye ST. Air pollutants, oxidative stress and human health. Mutat Res. (2009) 674:45–54. 10.1016/j.mrgentox.2008.10.005 19013537

[38] Solleiro-Villavicencio H Rivas-Arancibia S. Effect of chronic oxidative stress on neuroinflammatory response mediated by CD4(+)T cells in neurodegenerative diseases. Front Cell Neurosci. (2018) 12:114. 10.3389/fncel.2018.00114 29755324 PMC5934485

[39] Hahad O Lelieveld J Birklein F Lieb K Daiber A Munzel T. Ambient air pollution increases the risk of cerebrovascular and neuropsychiatric disorders through induction of inflammation and oxidative stress. Int J Mol Sci. (2020) 21:306. 10.3390/ijms21124306 32560306 PMC7352229

[40] Pope CA Bhatnagar A McCracken JP Abplanalp W Conklin DJ O'Toole T. Exposure to fine particulate air pollution is associated with endothelial injury and systemic inflammation. Circ Res. (2016) 119:1204–14. 10.1161/CIRCRESAHA.116.309279 27780829 PMC5215745

[41] Smith BJ Lerner MR Bu SY Lucas EA Hanas JS Lightfoot SA. Systemic bone loss and induction of coronary vessel disease in a rat model of chronic inflammation. Bone. (2006) 38:378–86. 10.1016/j.bone.2005.09.008 16256450

[42] Prada D Lopez G Solleiro-Villavicencio H Garcia-Cuellar C Baccarelli AA. Molecular and cellular mechanisms linking air pollution and bone damage. Environ Res. (2020) 185:109465. 10.1016/j.envres.2020.109465 32305664 PMC7430176

[43] Kitaura H Kimura K Ishida M Kohara H Yoshimatsu M Takano-Yamamoto T. Immunological reaction in TNF-alpha-mediated osteoclast formation and bone resorption in vitro and in vivo. Clin Dev Immunol. (2013) 2013:181849. 10.1155/2013/181849 23762085 PMC3676982

[44] O'Driscoll CA Gallo ME Fechner JH Schauer JJ Mezrich JD. Real-world PM extracts differentially enhance Th17 differentiation and activate the aryl hydrocarbon receptor (AHR). Toxicology. (2019) 414:14–26. 10.1016/j.tox.2019.01.002 30611761 PMC7065493

[45] Mousavi SE Amini H Heydarpour P Amini Chermahini F Godderis L. Air pollution, environmental chemicals, and smoking may trigger vitamin D deficiency: Evidence and potential mechanisms. Environ Int. (2019) 122:67–90. 10.1016/j.envint.2018.11.052 30509511

[46] He H Zeng Y Wang X Yang L Zhang M An Z. Meteorological condition and air pollution exposure associated with vitamin d deficiency: a cross-sectional population-based study in China. Risk Manag Healthc Policy. (2020) 13:2317–24. 10.2147/RMHP.S273145 33154683 PMC7605970

[47] Pacifici R. The role of IL-17 and TH17 cells in the bone catabolic activity of PTH. Front Immunol. (2016) 7:57. 10.3389/fimmu.2016.00057 26925062 PMC4756106

[48] Adamopoulos IE Chao CC Geissler R Laface D Blumenschein W Iwakura Y. Interleukin-17A upregulates receptor activator of NF-kappaB on osteoclast precursors. Arthritis Res Ther. (2010) 12:R29. 10.1186/ar2936 20167120 PMC2875663

[49] Yang C Li D Tian Y Wang P. Ambient air pollutions are associated with vitamin D status. Int J Environ Res Public Health. (2021) 18:887. 10.3390/ijerph18136887 34198962 PMC8297026

[50] Matsunawa M Amano Y Endo K Uno S Sakaki T Yamada S. The aryl hydrocarbon receptor activator benzo[a]pyrene enhances vitamin D3 catabolism in macrophages. Toxicol Sci. (2009) 109:50–8. 10.1093/toxsci/kfp044 19244278

[51] Manicourt DH Devogelaer JP. Urban tropospheric ozone increases the prevalence of vitamin D deficiency among Belgian postmenopausal women with outdoor activities during summer. J Clin Endocrinol Metab. (2008) 93:3893–9. 10.1210/jc.2007-2663 18628525

[52] Brown GC. Nitric oxide regulates mitochondrial respiration and cell functions by inhibiting cytochrome oxidase. FEBS Lett. (1995) 369:136–9. 10.1016/0014-5793(95)00763-Y 7649245

[53] Liu C Ma Q Liu Y Ma J He H. Synergistic reaction between SO_2_ and NO_2_ on mineral oxides: a potential formation pathway of sulfate aerosol. Phys Chem Chem Phys. (2012) 14:1668–76. 10.1039/C1CP22217A 21993907

[54] Kemp JP Morris JA Medina-Gomez C Forgetta V Warrington NM Youlten SE. Identification of 153 new loci associated with heel bone mineral density and functional involvement of GPC6 in osteoporosis. Nat Genet. (2017) 49:1468–75. 10.1038/ng.3949 28869591 PMC5621629

