# Supplementary material for: Air pollution, genetic factors and the risk of osteoporosis: A prospective study in the UK biobank
Source: Front Public Health. 2023 Mar 21;11:1119774. doi: 10.3389/fpubh.2023.1119774 (PMC10071034; doi:10.3389/fpubh.2023.1119774)
Supplement: Supplementary file 1 [file Data_Sheet_1.docx]

Supplementary file

# Supplementary tables and figures


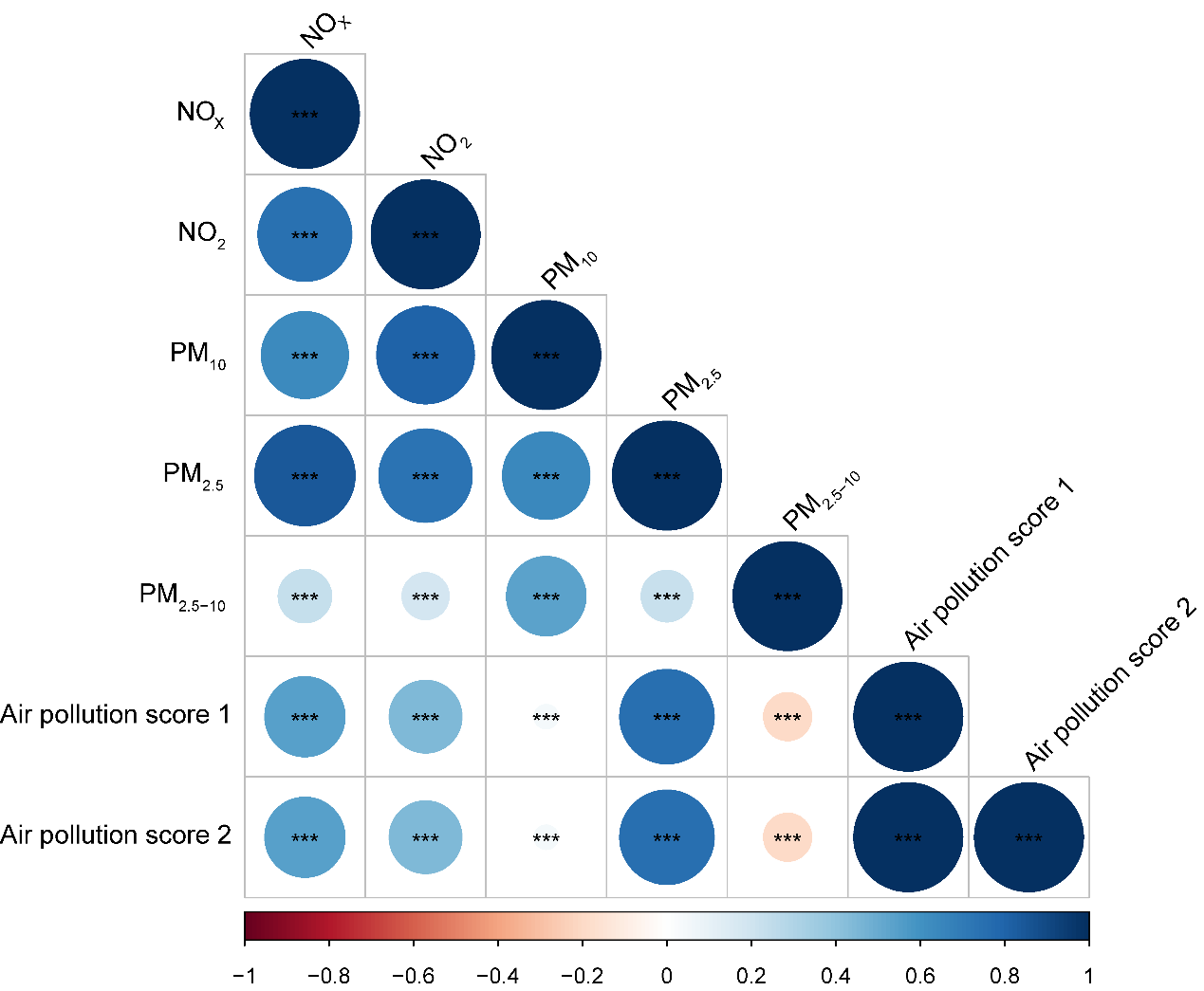


**Figure S1.** Correlation plots between different air pollutions and air pollution scores (APS). Note: Associations were adjusted for age, sex, genotyped batch, Townsend deprivation index, height, weight, smoking status. Abbreviations: PM_2.5_, particular matter with aerodynamic diameter ≤2.5μm; PM_10_, particular matter with an aerodynamic diameter ≤10μm; PM_2.5-10_, particular matter with an aerodynamic diameter between 2.5 and 10 μm; NO_2_, nitrogen dioxide; NO_x_, nitrogen oxides.


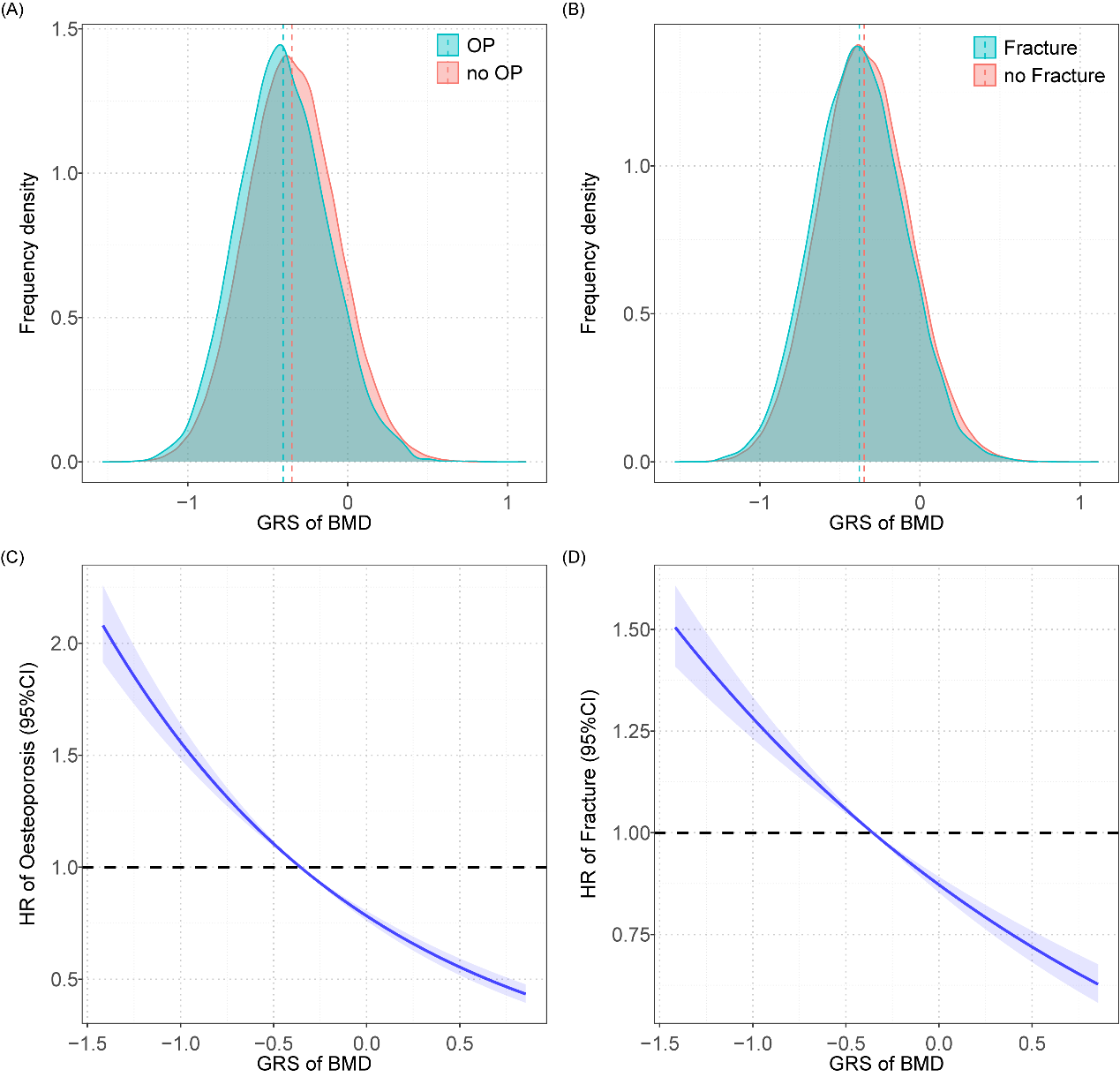
**Figure S2.** The distributions of sex specific GRS between cases and controls in UKB cohort. (**A**) Distributions of GRS between OP cases and controls; (**B**) Distributions of GRS between Fracture cases and controls; (**C**) Restricted cubic spline models for the relationship between GRS with osteoporosis risk; (**D**) Restricted cubic spline models for the relationship between GRS with fracture risk; Note: Associations were adjusted for age, sex, genotyped batch, Townsend deprivation index, height, weight, smoking status and the first 10 principal components of ancestry. Abbreviations: OP. osteoporosis CI, confidence interval; HR, hazard ratio; BMD, bone mineral density; GRS, genetic risk score.


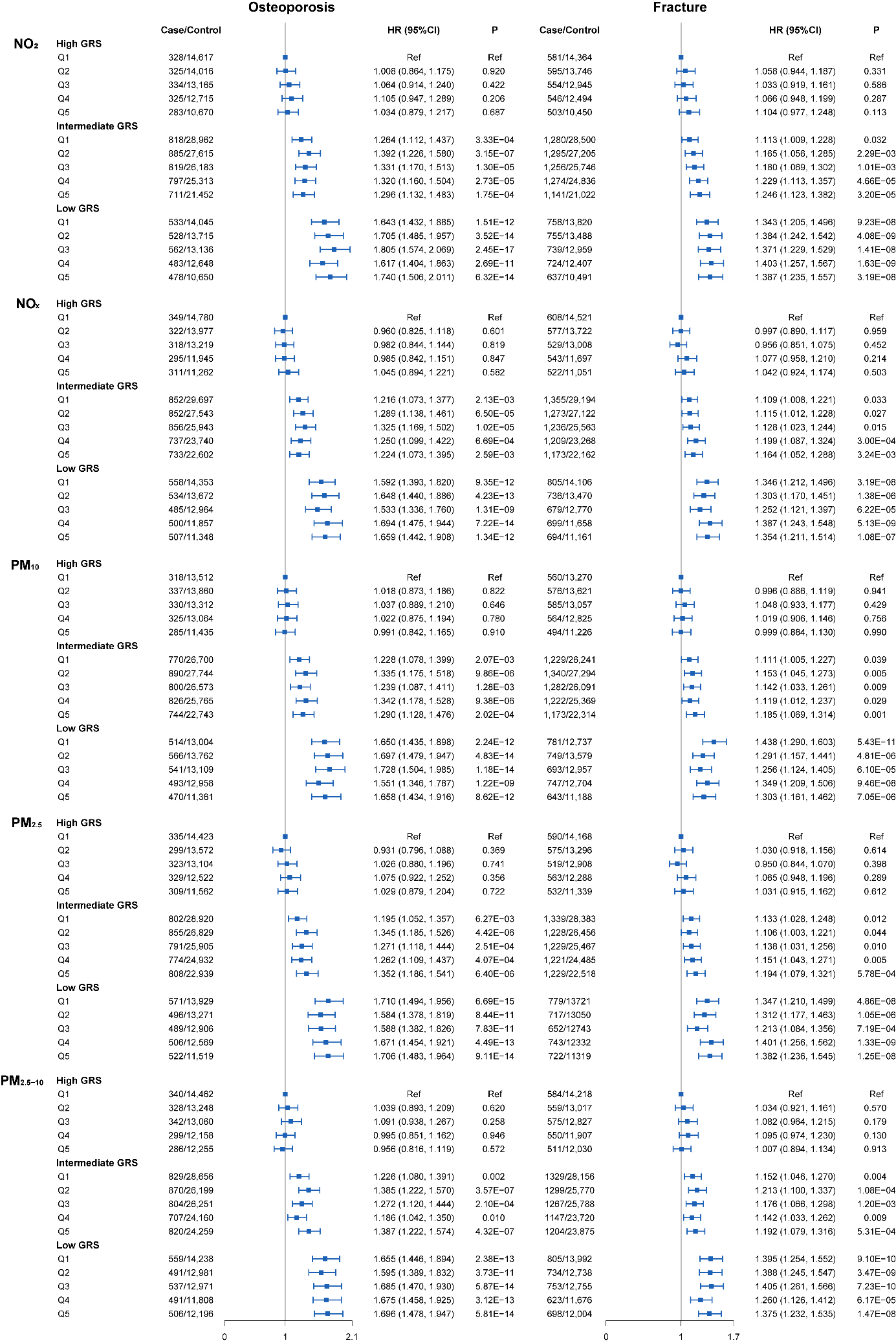


**Figure S3.** OP and fracture risks in the subgroups stratified by genetic risk and air pollutants’ concentrations (versus participants with lowest concentration of air pollutants in the highest genetic risk group) in the UKB cohort for each air pollutant. Note: Associations were adjusted for age, sex, genotyped batch, Townsend deprivation index, height, weight, smoking status and the first 10 principal components of ancestry. Abbreviations: CI, confidence interval; HR, hazard ratio; PM_2.5_, particular matter with aerodynamic diameter ≤2.5μm; PM_10_, particular matter with an aerodynamic diameter ≤10μm; PM_2.5-10_, particular matter with an aerodynamic diameter between 2.5 and 10μm; NO_2_, nitrogen dioxide; NO_x_, nitrogen oxides; GRS, genetic risk score.


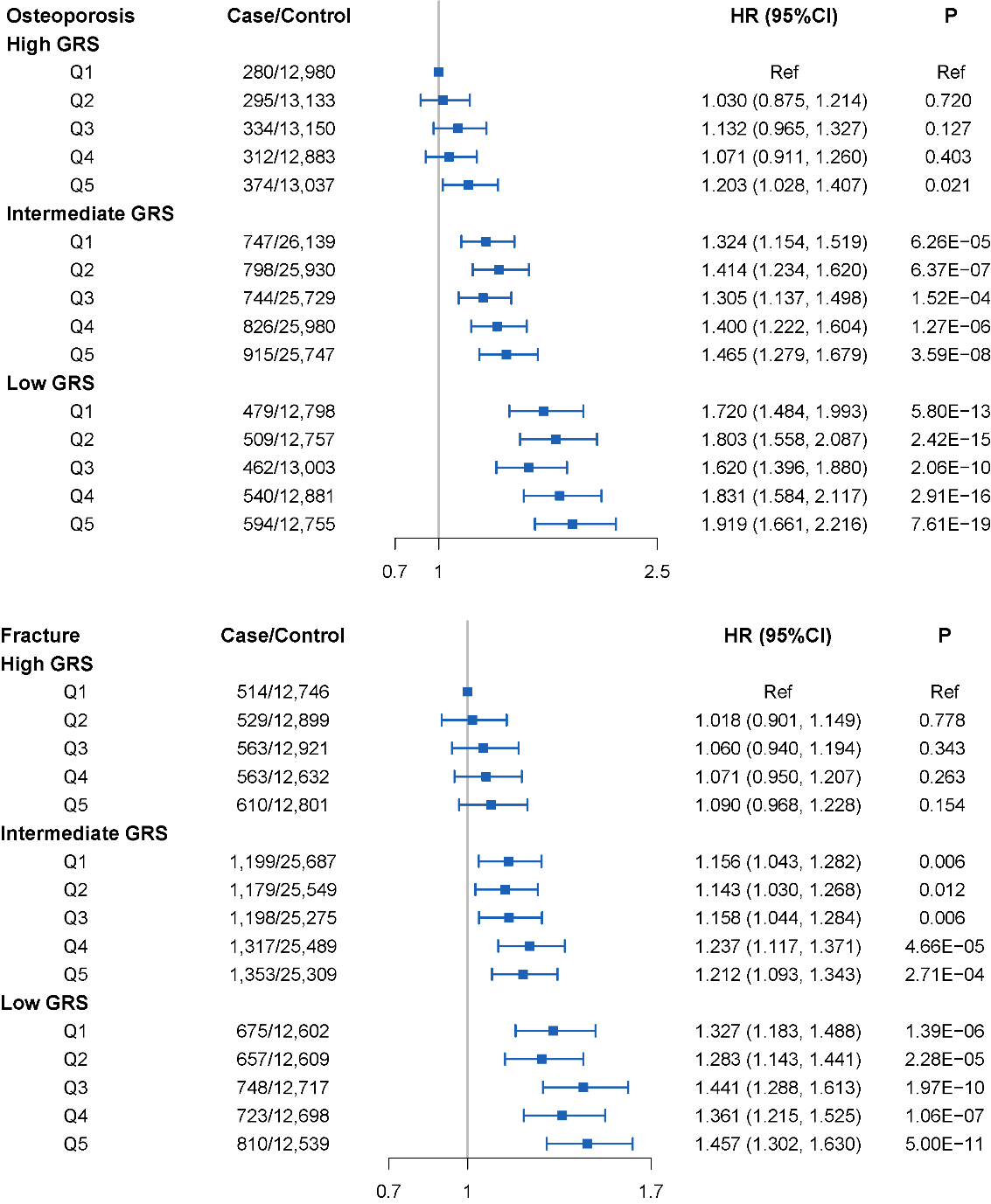


**Figure S4.** OP and fracture risks in the subgroups stratified by genetic risk and air pollution scores (APS_2_) concentrations (versus participants with lowest concentration of APS_2_ in the highest genetic risk group) in the UKB cohort. Note: Associations were adjusted for age, sex, genotyped batch, Townsend deprivation index, height, weight, smoking status and the first 10 principal components of ancestry. Abbreviations: CI, confidence interval; HR, hazard ratio; GRS, genetic risk score.

**Table S1. Literature review of the relationship between air pollution and OP in previous studies**

| **First author (Year)** | **Location** | **Sample Size** | **Study Design** | **Exposure** | **OP or Fracture Assessment** | **Conclusions** |
| --- | --- | --- | --- | --- | --- | --- |
| K. Alvær (2007) | Norway | 590 men (aged 75 to 76 years old) | Cross-sectional study | PM_2.5_, PM_10_, NO_2_ | Total body BMD by DXA Low BMD = BMD Z-score ≤ -1 | Total body BMD was inversely associated with both PM_2.5_ [β= -47, 95% CI= (-77, -17)] and PM_10_ [β= -28, 95% CI= (-48, -8)]. The risk of low total body BMD was positively associated with PM_2.5_ [OR= 1.33, 95% CI= (1.05, 1.70)] and PM_10_ [OR= 1.28, 95% CI= (1.00, 1.63)]. |
| Kuang-Hsi Chang (2015) | Taiwan, China | CO: 36608, NO_2_: 36561 (mean age 62.3 years) | Cohort study | CO, NO_2_ | OP was defined by the International Classification of Diseases, Ninth Revision, Clinical Modification | Compared to the first quartile group, exposure to the 4th quartile group of CO [HR = 1.84, 95% CI = (1.71, 1.98)] and NO_2_ [HR = 1.60, 95% CI = (1.48, 1.73)] was associated with an increased risk of OP. |
| R. Mazzucchelli (2018) | Spain | 4271 (mean age 83.8 years, 3346 women) | Cohort study | SO_2_, NO, NO_2_, O_3_, PM_2.5_, PM_10_ | Hip fracture record from the Hospital Universitario Fundacion Alcorcon database | Hip fracture incidence was associated with SO_2_ [IRR = 1.11, 95% CI = (1.04, 1.18)], NO [IRR = 1.01, 95% CI = (1.01, 1.02)], and NO2 [IRR = 1.02, 95% CI = (1.01, 1.04)] after adjusting for a natural spline function of time, season and mean air temperature. For O_3_, this association was negative [IRR = 0.97, 95% CI (0.95, 0.99)]. |
| Yu-Hsuan Lin (2020) | Taiwan, China | 4595 (mean age 49.7 ± 10.7 years, 2477 women) | Cross-sectional study | O_3_, CO, SO_2_, NO, NO_2_, NO_x_, PM_2.5_, PM_10_ | BMD T-score of calcaneus in the non-dominant foot between -1.0 and -2.5 was defined as osteopenia, <-2.5 was defined as OP | O_3_ (β = 0.015; p = 0.008) was significantly positively associated with T-score, CO (β = -0.809; p < 0.001), SO_2_ (β = -0.050; p = 0.005), NO (β = -0.040; p < 0.001), NO2 (β = -0.023; p < 0.001), and NO_x_ (β = -0.017; p < 0.001) were significantly negatively associated with T-score after multivariable adjustments. There were not significantly association between T-score and PM_2.5_/PM_10_. |
| Jung Hun Sung (2020) | Korea | 44602 women (aged over 50 years) | Cohort study | PM_2.5_, PM_10_, PM_2.5-10_ | OFs were defined as having one hospitalization or two physician visits within 6 month under any fracture diagnosis code | After adjusting for age, household income, Charlson Comorbidity Index and region, PM_2.5_ increased the risk of OF (4th quartile group aHR = 1.13, 95% CI 1.02-1.24), spine and non-spine fractures [4th quartile group aHR = 1.17, 95% CI = (1.00, 1.38); aHR = 1.16, 95% CI = (1.01, 1.33)] compared to the first quartile group. There was no association between PM_10_/PM_2.5-10_ and OF. |
| Otavio T. Ranzani (2020) | India | 3717 (mean age 35.7 years, 1711 women) | Cross-sectional study | PM_2.5_, BC | BMC and BMD corrected by bone area at the lumbar spine and left hip measured by DXA | In fully adjusted models, PM_2.5_ (per 3μg/m^3^ increase) was associated with BMC in the spine [mean difference = -0.57; 95 % CI = (-1.06, -0.07)] and hip [mean difference = -0.13; 95% CI = (-0.3, 0.03)]. After confounder adjustment, PM_2.5_ (per 3μg/m^3^ increase) was also associated with BMD in the spine [mean difference = -0.011; 95% CI = (-0.021, 0)] and hip [mean difference = -0.004; 95% CI = (-0.008, 0.001)]. Exposure to BC (per 1μg/m^3^ increase) was associated with lower BMC in the spine [mean difference = -1.13; 95% CI = (-2.81, 0.54)] and hip [mean difference = -0.35; 95% CI = (-0.96, 0.25)]. There was no association between biomass fuel use and spine BMC. |
| Dou Qiao (2020) | China | 8033 (aged 18 to 79 years, 5032 women) | Cross-sectional study | PM_1_, PM_2.5_, PM_10_ and NO_2_ | OP was defined based on the BMD T-score ≤ -2.5 | OP was associated with PM_1_ [OR = 2.08, 95% CI = (1.72, 2.50)], PM_2.5_ [OR=2.28, 95% CI = (1.90, 2.74)], PM_10_ [OR = 1.93, 95% CI = (1.60, 2.32)] and NO_2_ [OR = 2.02, 95% CI = (1.68, 2.41)]. Per 1μg/m^3^ increase in PM_1_, PM_2.5_, PM_10_ and NO_2_ were associated with a 14.9%, 14.6%, 7.3%, and 16.5% elevated risk of OP. |
| Jinyoung Shin (2021) | Korea | 237149 (aged ≥40 years, 122991 women) | Cohort study | PM_2.5_, PM_10_, CO, SO_2_ and NO_2_ | OP was defined as claim codes and prescriptions of bisphosphonates or selective estrogen receptor modulators at least twice annually | Exposure to PM_10_ was positively associated with incidence of OP [4th quartile group HR = 1.034, 95% CI = (1.009-1.062)] compared to the first quartile group. However, there was no increase in OP based on exposure to NO_2_, CO, SO_2_ or PM_2.5_. |
| G. Adami (2021) | Italy | 59950 women (mean age 65.1 years) | Cohort study | PM_2.5_, PM_10_ | OP was defined based on the T-score at any site < -2.5 | PM_2.5_ exposure was negatively associated with T-score levels at the femoral neck [β = -0.005, 95 CI = (-0.007, -0.003)] and lumbar spine [β = -0.003, 95% CI = (-0.006, -0.001)]. |
| Jialong Wu (2021) | China | 66598 (aged 30 to 79 ) | Cohort study | PM_1_, PM_2.5_, PM_10_, NO_2_ | Bone strength was expressed by the calcaneus QUI measured by quantitative ultrasound, with higher QUI values indicating greater bone strength | PM_1_, PM_2.5_, PM_10_, and NO_2_ (per 10μg/m^3^ increase) were negatively associated with QUI, mean difference (95% CI) was [-5.38, (-6.17, -4.60), [-1.89, (-2.33, -1.44)], [-0.77, (-1.08, -0.47) and [-2.02, (-2.32, -1.71)], respectively, after adjusting for diet variables, ultraviolet radiation, occupation, and indoor heating use. |

Abbreviations: OP, osteoporosis; OF, osteoporotic fracture; O_3_, ozone; NO_x_, nitrogen oxides; CO, carbon monoxide; NO_2_, nitrogen dioxide; SO_2_, sulfur dioxide; NO, nitrogen oxide; PM_2.5_, particular matter with an aerodynamic diameter ≤2.5μm; PM_10_, particular matter with an aerodynamic diameter ≤10μm; PM_2.5-10_, particular matter with an aerodynamic diameter between 2.5 and 10μm; PM_1_, particulate matter with an aerodynamic diameter ≤1.0μm; BC, black carbon; BMC, bone mineral content; BMD, bone mineral density; DXA, dual-energy x-ray absorptiometry; HR, hazard ratio; CI, confidence interval; IRR, incidence rate ratio; QUI, quantitative ultrasound index.

**Table S2. Associations between GRSs in different cutoffs and OP risks**

| **Variables** | **Cutoffs** | **Estimate (95% CI)** | ***P*** |
| --- | --- | --- | --- |
| eBMD | <5×10^-4^ | 0.015 (0.013, 0.016) | 9.38E-49 |
|  | <5×10^-5^ | 0.032 (0.028, 0.035) | 4.88E-66 |
|  | **<5×10^-6^** | **0.050 (0.045, 0.055)** | **6.08E-83** |
|  | <5×10^-7^ | 0.051 (0.044, 0.057) | 2.33E-54 |
|  | <5×10^-8^ | 0.078 (0.063, 0.094) | 9.37E-23 |
| OP* | <5×10^-4^ | -0.222 (-1.792, 1.347) | 4.25E-07 |
|  | <5×10^-5^ | -0.450 (-1.700, 0.800) | 5.05E-08 |
|  | **<5×10^-6^** | **-0.765 (-1.677, 0.147)** | **7.27E-11** |
|  | <5×10^-7^ | -0.794 (-1.680, 0.092) | 6.85E-08 |
|  | <5×10^-8^ | -0.831 (-1.685, 0.022) | 1.99E-02 |
| Fracture* | <5×10^-4^ | -0.095 (-1.877, 1.687) | 0.009 |
|  | <5×10^-5^ | -0.215 (-1.796, 1.367) | 0.002 |
|  | **<5×10^-6^** | **-0.295 (-1.754, 1.164)** | **0.002** |
|  | <5×10^-7^ | -0.294 (-1.755, 1.168) | 0.015 |
|  | <5×10^-8^ | -0.760 (-1.677, 0.157) | 0.010 |

Note: Associations were adjusted for age, sex, genotyped batch, Townsend deprivation index, height, weight, smoking status and the first 10 principal components of ancestry. *OR was estimated for associations between GRS in different cut-offs and OP/fracture risk. Abbreviations: eBMD, estimated bone mineral density; OP, osteoporosis; CI, confidence interval; OR, odds ratio; GRS, genetic risk score. Bold and highlighted red text indicates the chosen cut-off values for the construction of GRS.

**Table S3. Summary results of Femoral-Neck bone mineral density associated SNPs used for genetic risk score.**

| **Chromosome** | **rsID** | **Position** | **Allele A** | **Allele B** | **Beta** | **Standard error** | **Z** | ***P*** |
| --- | --- | --- | --- | --- | --- | --- | --- | --- |
| 1 | rs4240913 | 10,437,804 | G | A | 0.053 | 0.011 | 4.673 | 4.61E-06 |
| 1 | rs3971300 | 22,484,575 | C | T | -0.041 | 0.009 | -4.759 | 3.27E-06 |
| 1 | rs113784679 | 22,648,479 | T | G | 0.098 | 0.020 | 4.807 | 2.60E-06 |
| 1 | rs7524102 | 22,698,447 | G | A | 0.084 | 0.010 | 8.531 | 7.36E-17 |
| 1 | rs6588304 | 68,631,356 | T | C | -0.040 | 0.008 | -5.234 | 3.10E-07 |
| 1 | rs76962711 | 68,641,930 | A | G | 0.084 | 0.016 | 5.205 | 3.59E-07 |
| 1 | rs2566752 | 68,656,697 | C | T | 0.062 | 0.008 | 8.045 | 3.65E-15 |
| 1 | rs12402966 | 172,203,894 | A | G | 0.054 | 0.010 | 5.575 | 5.00E-08 |
| 1 | rs10924510 | 236,336,801 | G | C | 0.070 | 0.015 | 4.757 | 3.30E-06 |
| 2 | rs72794538 | 42,281,656 | T | G | 0.051 | 0.010 | 5.169 | 4.33E-07 |
| 2 | rs2941584 | 54,881,621 | T | C | -0.039 | 0.008 | -4.800 | 2.68E-06 |
| 2 | rs55983207 | 119,529,829 | C | T | 0.101 | 0.019 | 5.274 | 2.52E-07 |
| 2 | rs138243057 | 141,984,025 | C | T | 0.329 | 0.068 | 4.807 | 2.60E-06 |
| 2 | rs10170839 | 166,572,906 | C | A | -0.059 | 0.008 | -7.892 | 1.20E-14 |
| 2 | rs13015453 | 202,805,458 | T | C | -0.037 | 0.008 | -4.716 | 4.01E-06 |
| 3 | rs436448 | 41,121,251 | T | C | -0.064 | 0.008 | -8.439 | 1.56E-16 |
| 3 | rs112196420 | 69,499,803 | T | G | 0.102 | 0.021 | 4.787 | 2.86E-06 |
| 4 | rs35654957 | 1,010,077 | C | T | -0.045 | 0.008 | -5.448 | 1.00E-07 |
| 4 | rs1381635 | 88,587,359 | G | A | -0.124 | 0.026 | -4.847 | 2.15E-06 |
| 4 | rs12505482 | 88,730,210 | C | T | -0.042 | 0.008 | -5.198 | 3.73E-07 |
| 4 | rs75393680 | 143,686,326 | G | A | 0.093 | 0.019 | 4.977 | 1.14E-06 |
| 5 | rs163358 | 16,424,194 | C | T | 0.058 | 0.012 | 4.736 | 3.64E-06 |
| 5 | rs1366594 | 88,376,061 | C | A | -0.079 | 0.008 | -10.559 | 5.44E-25 |
| 5 | rs72792012 | 126,863,645 | C | G | 0.192 | 0.039 | 4.875 | 1.87E-06 |
| 5 | rs12332505 | 130,337,348 | G | T | 0.043 | 0.009 | 4.739 | 3.58E-06 |
| 5 | rs12521108 | 135,409,124 | A | G | 0.035 | 0.008 | 4.694 | 4.44E-06 |
| 6 | rs10946458 | 21,391,282 | C | T | -0.045 | 0.008 | -5.633 | 3.63E-08 |
| 6 | rs13194508 | 127,144,683 | C | T | -0.052 | 0.009 | -5.815 | 1.30E-08 |
| 6 | rs1832727 | 151,857,713 | C | G | -0.054 | 0.008 | -7.103 | 3.34E-12 |
| 6 | rs1023940 | 151,932,778 | A | G | 0.045 | 0.008 | 6.034 | 3.64E-09 |
| 6 | rs1609624 | 152,081,009 | T | C | -0.038 | 0.008 | -4.800 | 2.68E-06 |
| 6 | rs76784590 | 170,411,450 | A | G | 0.111 | 0.024 | 4.695 | 4.42E-06 |
| 7 | rs11551167 | 2,394,991 | C | T | -0.193 | 0.006 | -33.483 | 1.45E-236 |
| 7 | rs28362721 | 30,957,702 | T | C | -0.049 | 0.010 | -4.937 | 1.39E-06 |
| 7 | rs4281029 | 38,153,650 | A | C | 0.057 | 0.009 | 6.069 | 2.96E-09 |
| 7 | rs4448201 | 96,154,912 | G | C | -0.066 | 0.008 | -8.313 | 4.37E-16 |
| 7 | rs3779381 | 120,966,790 | G | A | 0.058 | 0.009 | 6.805 | 2.87E-11 |
| 8 | rs4517154 | 72,393,336 | G | T | 0.039 | 0.007 | 5.204 | 3.61E-07 |
| 8 | rs1485307 | 120,007,395 | T | C | 0.062 | 0.008 | 8.094 | 2.49E-15 |
| 8 | rs67282053 | 121,028,220 | T | C | -0.038 | 0.008 | -4.698 | 4.35E-06 |
| 9 | rs74464964 | 89,824,970 | A | G | 0.074 | 0.015 | 4.871 | 1.91E-06 |
| 9 | rs17194561 | 109,522,849 | G | A | -0.086 | 0.017 | -5.108 | 5.52E-07 |
| 9 | rs7850979 | 110,920,305 | A | G | 0.043 | 0.009 | 4.928 | 1.45E-06 |
| 9 | rs10817222 | 114,596,299 | T | A | -0.044 | 0.009 | -4.777 | 3.00E-06 |
| 9 | rs9657746 | 133,481,563 | G | C | -0.041 | 0.008 | -5.214 | 3.43E-07 |
| 10 | rs11195150 | 112,240,383 | T | C | 0.052 | 0.011 | 4.730 | 3.75E-06 |
| 10 | rs2292626 | 124,186,714 | T | C | -0.039 | 0.008 | -5.222 | 3.07E-07 |
| 11 | rs7108738 | 15,710,084 | G | T | 0.083 | 0.010 | 8.520 | 8.07E-17 |
| 11 | rs10832587 | 16,304,261 | A | T | 0.040 | 0.008 | 5.238 | 3.04E-07 |
| 11 | rs11024028 | 16,756,873 | G | C | 0.056 | 0.010 | 5.725 | 2.18E-08 |
| 11 | rs1785493 | 68,208,345 | T | C | -0.045 | 0.008 | -5.613 | 4.06E-08 |
| 11 | rs3918298 | 69,463,273 | A | G | -0.110 | 0.023 | -4.717 | 3.99E-06 |
| 12 | rs79881709 | 28,019,024 | A | G | -0.052 | 0.010 | -5.096 | 6.27E-07 |
| 12 | rs4759086 | 53,666,713 | A | G | 0.040 | 0.008 | 5.035 | 7.99E-07 |
| 12 | rs4759320 | 54,433,011 | C | G | -0.045 | 0.008 | -5.649 | 3.33E-08 |
| 13 | rs7992415 | 42,975,297 | C | A | 0.089 | 0.016 | 5.404 | 1.27E-07 |
| 14 | rs73328524 | 91,496,078 | G | C | 0.050 | 0.010 | 4.922 | 1.49E-06 |
| 15 | rs7173595 | 51,533,736 | C | T | -0.037 | 0.008 | -4.746 | 3.48E-06 |
| 15 | rs4776341 | 67,414,911 | G | T | 0.038 | 0.008 | 4.989 | 1.07E-06 |
| 16 | rs10794639 | 377,367 | G | A | -0.051 | 0.008 | -6.784 | 3.30E-11 |
| 16 | rs8046561 | 1,526,251 | C | G | -0.040 | 0.008 | -5.206 | 3.57E-07 |
| 16 | rs147501507 | 48,094,897 | T | C | -0.125 | 0.027 | -4.699 | 4.34E-06 |
| 16 | rs2047937 | 49,864,791 | C | T | 0.039 | 0.007 | 5.308 | 2.10E-07 |
| 16 | rs62028332 | 51,025,468 | A | G | 0.060 | 0.012 | 5.239 | 3.01E-07 |
| 16 | rs71390846 | 86,714,715 | C | G | -0.059 | 0.010 | -6.058 | 3.16E-09 |
| 17 | rs7209460 | 2,048,713 | C | T | -0.051 | 0.008 | -6.199 | 1.35E-09 |
| 17 | rs2741856 | 41,826,839 | C | G | 0.088 | 0.014 | 6.201 | 1.34E-09 |
| 17 | rs117697841 | 42,207,547 | A | G | 0.082 | 0.013 | 6.275 | 8.50E-10 |
| 17 | rs9906807 | 42,303,305 | T | C | 0.043 | 0.008 | 5.069 | 7.17E-07 |
| 17 | rs9912123 | 64,411,964 | A | G | 0.159 | 0.033 | 4.859 | 2.02E-06 |
| 17 | rs4387633 | 69,927,592 | G | A | 0.036 | 0.007 | 4.816 | 2.48E-06 |
| 18 | rs884205 | 60,054,857 | A | C | -0.042 | 0.009 | -4.799 | 2.71E-06 |
| 19 | rs12460195 | 33,584,479 | A | G | 0.042 | 0.009 | 4.872 | 1.78E-06 |
| 20 | rs73100693 | 14,624,598 | A | T | 0.049 | 0.010 | 5.007 | 9.80E-07 |
| 22 | rs2267000 | 23,455,366 | T | C | 0.036 | 0.008 | 4.726 | 3.82E-06 |
| 22 | rs143936251 | 47,092,139 | T | C | -0.071 | 0.015 | -4.740 | 3.58E-06 |

**Table S4. Linear relationship between air pollutants and eBMD**

|  | **Beta (95%CI)** | ***P*** | ***P _Bonferroni_*** |
| --- | --- | --- | --- |
| NO_2_ | -0.010 (-0.013, -0.007) | 2.78E-10 | 1.39E-09 |
| NO_x_ | -0.010 (-0.013, -0.007) | 1.29E-08 | 6.45E-08 |
| PM_10_ | -0.019 (-0.022, -0.016) | 6.32E-31 | 3.16E-30 |
| PM_2.5_ | -0.009 (-0.012, -0.005) | 1.73E-07 | 8.65E-07 |
| PM_2.5-10_ | -0.003 (-0.006, 0.000) | 0.029 | 0.145 |

Note: Associations were adjusted for age, sex, genotyped batch, Townsend deprivation index, height, weight, smoking status. Abbreviations: CI, confidence interval; PM_2.5_, particular matter with aerodynamic diameter ≤2.5μm; PM_10_, particular matter with an aerodynamic diameter ≤10μm; PM_2.5-10_, particular matter with an aerodynamic diameter between 2.5 and 10μm; NO_2_, nitrogen dioxide; NO_x_, nitrogen oxides.

**Table S5. Linear relationship between air pollutants and eBMD (sensitivity analysis)**

|  | **Beta (95%CI)** | ***P*** | ***P _Bonferroni_*** |
| --- | --- | --- | --- |
| NO_2_ | -0.009 (-0.013, -0.006) | 2.88E-08 | 1.44E-07 |
| NO_x_ | -0.011 (-0.015, -0.007) | 9.81E-10 | 4.90E-09 |
| PM_10_ | -0.021 (-0.024, -0.017) | 1.25E-34 | 6.24E-34 |
| PM_2.5_ | -0.007 (-0.011, -0.004) | 1.15E-05 | 5.77E-05 |
| PM_2.5-10_ | -0.004 (-0.007, -0.001) | 0.015 | 0.075 |

Note: Associations were adjusted for batch, centre, age, sex, race, Townsend deprivation index, height, weight, smoking status, alcohol, physical activity, diet, CKD, T2D, cancer, and deprivation. Abbreviations: CI, confidence interval; PM_2.5_, particular matter with aerodynamic diameter ≤2.5μm; PM_10_, particular matter with an aerodynamic diameter ≤10μm; PM_2.5-10_, particular matter with an aerodynamic diameter between 2.5 and 10μm; NO_2_, nitrogen dioxide; NO_x_, nitrogen oxides; CKD, chronic kidney disease; T2D, Type 2 diabetes.

**Table S6. The 95% confidence interval and adjusted hazard ratio for individual air pollutant concentration with the risk of osteoporosis in the UK Biobank study**

|  |  | **HR (95% CI)** | **Case/Control** | ***P*** |
| --- | --- | --- | --- | --- |
| PM_2.5_ | Q1 | 1 | 1708/57272 |  |
|  | Q2 | 1.034 (0.979, 1.093) | 1650/53672 | 0.230 |
|  | Q3 | 1.030 (0.974, 1.089) | 1603/51915 | 0.297 |
|  | Q4 | 1.083 (1.024, 1.145) | 1609/50023 | 0.005 |
|  | Q5 | 1.129 (1.065, 1.197) | 1639/46020 | 4.59E-05 |
|  | Linear | 1.046 (1.027, 1.066) |  | 2.31E-06 |
| PM_10_ | Q1 | 1 | 1602/53216 |  |
|  | Q2 | 1.054 (0.997, 1.114) | 1793/55366 | 0.063 |
|  | Q3 | 1.052 (0.995, 1.113) | 1671/52994 | 0.073 |
|  | Q4 | 1.050 (0.993, 1.110) | 1644/51787 | 0.088 |
|  | Q5 | 1.040 (0.980, 1.103) | 1499/45539 | 0.197 |
|  | Linear | 1.009 (0.990, 1.028) | / | 0.357 |
| PM_2.5-10_ | Q1 | 1 | 1728/57356 |  |
|  | Q2 | 1.034 (0.980, 1.091) | 1689/52428 | 0.224 |
|  | Q3 | 1.049 (0.995, 1.107) | 1683/52282 | 0.078 |
|  | Q4 | 1.001 (0.947, 1.057) | 1497/48126 | 0.976 |
|  | Q5 | 1.051 (0.995, 1.109) | 1612/48710 | 0.073 |
|  | Linear | 1.007 (0.990, 1.024) | / | 0.431 |
| NO_2_ | Q1 | 1 | 1679/57624 |  |
|  | Q2 | 1.079 (1.021, 1.140) | 1738/55346 | 0.007 |
|  | Q3 | 1.101 (1.041, 1.163) | 1715/52484 | 6.74E-04 |
|  | Q4 | 1.107 (1.047, 1.172) | 1605/50676 | 4.03E-04 |
|  | Q5 | 1.095 (1.030, 1.164) | 1472/42772 | 0.004 |
|  | Linear | 1.029 (1.008, 1.049) | / | 0.005 |
| NO_x_ | Q1 | 1 | 1759/58830 |  |
|  | Q2 | 1.058 (1.003, 1.118) | 1708/55192 | 0.040 |
|  | Q3 | 1.068 (1.011, 1.128) | 1659/52126 | 0.020 |
|  | Q4 | 1.081 (1.021, 1.144) | 1532/47542 | 0.007 |
|  | Q5 | 1.087 (1.025, 1.154) | 1551/45212 | 0.006 |
|  | Linear | 1.029 (1.011, 1.048) | / | 0.002 |

Note: Associations were adjusted for age, sex, genotyped batch, Townsend deprivation index, height, weight, smoking status. Abbreviations: CI, confidence interval; HR, hazard ratio; PM_2.5_, particular matter with aerodynamic diameter ≤2.5μm; PM_10_, particular matter with an aerodynamic diameter ≤10μm; PM_2.5-10_, particular matter with an aerodynamic diameter between 2.5 and 10μm; NO_2_, nitrogen dioxide; NO_x_, nitrogen oxides.

**Table S7. The 95% confidence interval and adjusted hazard ratio for individual air pollutant concentration with the risk of osteoporosis in the UK Biobank study (sensitivity analysis)**

|  |  | **HR (95% CI)** | **Case/Control** | ***P*** |
| --- | --- | --- | --- | --- |
| PM_2.5_ | Q1 | 1 | 1708/57272 |  |
|  | Q2 | 1.010 (0.954, 1.068) | 1650/53672 | 0.739 |
|  | Q3 | 0.997 (0.941, 1.055) | 1603/51915 | 0.908 |
|  | Q4 | 1.041 (0.983, 1.103) | 1609/50023 | 0.170 |
|  | Q5 | 1.083 (1.019, 1.151) | 1639/46020 | 0.010 |
|  | Linear | 1.032 (1.012, 1.052) |  | 0.002 |
| PM_10_ | Q1 | 1 | 1602/53216 |  |
|  | Q2 | 1.037 (0.980, 1.098) | 1793/55366 | 0.212 |
|  | Q3 | 1.027 (0.969, 1.087) | 1671/52994 | 0.373 |
|  | Q4 | 1.036 (0.978, 1.097) | 1644/51787 | 0.230 |
|  | Q5 | 1.053 (0.990, 1.120) | 1499/45539 | 0.100 |
|  | Linear | 1.016 (0.996, 1.036) | / | 0.115 |
| PM_2.5-10_ | Q1 | 1 | 1728/57356 |  |
|  | Q2 | 1.021 (0.966, 1.079) | 1689/52428 | 0.467 |
|  | Q3 | 1.046 (0.990, 1.106) | 1683/52282 | 0.112 |
|  | Q4 | 0.997 (0.942, 1.055) | 1497/48126 | 0.915 |
|  | Q5 | 1.049 (0.992, 1.110) | 1612/48710 | 0.094 |
|  | Linear | 1.008 (0.991, 1.026) | / | 0.362 |
| NO_2_ | Q1 | 1 | 1679/57624 |  |
|  | Q2 | 1.056 (0.998, 1.118) | 1738/55346 | 0.058 |
|  | Q3 | 1.069 (1.010, 1.132) | 1715/52484 | 0.022 |
|  | Q4 | 1.058 (0.997, 1.121) | 1605/50676 | 0.061 |
|  | Q5 | 1.089 (1.022, 1.161) | 1472/42772 | 0.008 |
|  | Linear | 1.035 (1.013, 1.056) | / | 0.001 |
| NO_x_ | Q1 | 1 | 1759/58830 |  |
|  | Q2 | 1.041 (0.984, 1.100) | 1708/55192 | 0.163 |
|  | Q3 | 1.034 (0.977, 1.094) | 1659/52126 | 0.249 |
|  | Q4 | 1.037 (0.978, 1.100) | 1532/47542 | 0.225 |
|  | Q5 | 1.060 (0.997, 1.127) | 1551/45212 | 0.064 |
|  | Linear | 1.022 (1.003, 1.042) | / | 0.024 |

Note: Associations were adjusted for batch, centre, age, sex, race, Townsend deprivation index, height, weight, smoking status, alcohol, physical activity, diet, CKD, T2D, cancer, and deprivation. Abbreviations: CI, confidence interval; HR, hazard ratio; PM_2.5_, particular matter with aerodynamic diameter ≤2.5μm; PM_10_, particular matter with an aerodynamic diameter ≤10μm; PM_2.5-10_, particular matter with an aerodynamic diameter between 2.5 and 10μm; NO_2_, nitrogen dioxide; NO_x_, nitrogen oxides; CKD, chronic kidney disease; T2D, Type 2 diabetes.

**Table S8. The 95% confidence interval and adjusted hazard ratio for individual air pollutant concentration with the risk of fracture in the UK Biobank study**

|  |  | **HR (95% CI)** | **Case/Control** | ***P*** |
| --- | --- | --- | --- | --- |
| PM_2.5_ | Q1 | 1 | 1708/57272 |  |
|  | Q2 | 1.011 (0.967, 1.057) | 1650/53672 | 0.642 |
|  | Q3 | 0.974 (0.931, 1.019) | 1603/51915 | 0.246 |
|  | Q4 | 1.027 (0.982, 1.075) | 1609/50023 | 0.242 |
|  | Q5 | 1.050 (1.001, 1.102) | 1639/46020 | 0.043 |
|  | Linear | 1.018 (1.003, 1.034) | / | 0.020 |
| PM_10_ | Q1 | 1 | 1602/53216 |  |
|  | Q2 | 0.982 (0.939, 1.027) | 1793/55366 | 0.430 |
|  | Q3 | 0.980 (0.937, 1.025) | 1671/52994 | 0.381 |
|  | Q4 | 0.986 (0.942, 1.031) | 1644/51787 | 0.531 |
|  | Q5 | 0.965 (0.919, 1.012) | 1499/45539 | 0.143 |
|  | Linear | 0.987 (0.971, 1.002) | / | 0.087 |
| PM_2.5-10_ | Q1 | 1 | 1728/57356 |  |
|  | Q2 | 1.010 (0.966, 1.055) | 1689/52428 | 0.666 |
|  | Q3 | 1.018 (0.975, 1.064) | 1683/52282 | 0.414 |
|  | Q4 | 0.973 (0.930, 1.017) | 1497/48126 | 0.228 |
|  | Q5 | 1.001 (0.958, 1.047) | 1612/48710 | 0.962 |
|  | Linear | 0.994 (0.980, 1.009) | / | 0.433 |
| NO_2_ | Q1 | 1 | 1679/57624 |  |
|  | Q2 | 1.040 (0.994, 1.087) | 1738/55346 | 0.089 |
|  | Q3 | 1.043 (0.996, 1.091) | 1715/52484 | 0.071 |
|  | Q4 | 1.082 (1.034, 1.133) | 1605/50676 | 0.001 |
|  | Q5 | 1.078 (1.025, 1.133) | 1472/42772 | 0.003 |
|  | Linear | 1.022 (1.005, 1.039) | / | 0.009 |
| NO_x_ | Q1 | 1 | 1759/58830 |  |
|  | Q2 | 1.016 (0.972, 1.061) | 1708/55192 | 0.490 |
|  | Q3 | 0.991 (0.947, 1.037) | 1659/52126 | 0.690 |
|  | Q4 | 1.053 (1.006, 1.103) | 1532/47542 | 0.027 |
|  | Q5 | 1.042 (0.993, 1,094) | 1551/45212 | 0.095 |
|  | Linear | 1.023 (1.007, 1.038) | / | 0.003 |

Note: Associations were adjusted for age, sex, genotyped batch, Townsend deprivation index, height, weight, smoking status. Abbreviations: CI, confidence interval; HR, hazard ratio; PM_2.5_, particular matter with aerodynamic diameter ≤2.5μm; PM_10_, particular matter with an aerodynamic diameter ≤10μm; PM_2.5-10_, particular matter with an aerodynamic diameter between 2.5 and 10μm; NO_2_, nitrogen dioxide; NO_x_, nitrogen oxides.

**Table S9. The 95% confidence interval and adjusted hazard ratio for individual air pollutant concentration with the risk of fracture in the UK Biobank study (sensitivity analysis)**

|  |  | **HR (95% CI)** | **Case/Control** | ***P*** |
| --- | --- | --- | --- | --- |
| PM_2.5_ | Q1 | 1 | 1708/57272 |  |
|  | Q2 | 0.997 (0.953, 1.044) | 1650/53672 | 0.898 |
|  | Q3 | 0.949 (0.906, 0.995) | 1603/51915 | 0.028 |
|  | Q4 | 1.004 (0.958, 1.052) | 1609/50023 | 0.869 |
|  | Q5 | 1.024 (0.975, 1.076) | 1639/46020 | 0.344 |
|  | Linear | 1.009 (0.993, 1.025) | / | 0.275 |
| PM_10_ | Q1 | 1 | 1602/53216 |  |
|  | Q2 | 0.974 (0.930, 1.020) | 1793/55366 | 0.256 |
|  | Q3 | 0.962 (0.918, 1.008) | 1671/52994 | 0.102 |
|  | Q4 | 0.977 (0.932, 1.023) | 1644/51787 | 0.317 |
|  | Q5 | 0.964 (0.917, 1.013) | 1499/45539 | 0.149 |
|  | Linear | 0.989 (0.973, 1.005) | / | 0.188 |
| PM_2.5-10_ | Q1 | 1 | 1728/57356 |  |
|  | Q2 | 1.007 (0.963, 1.054) | 1689/52428 | 0.754 |
|  | Q3 | 1.011 (0.966, 1.058) | 1683/52282 | 0.634 |
|  | Q4 | 0.976 (0.931, 1.022) | 1497/48126 | 0.297 |
|  | Q5 | 0.994 (0.950, 1.041) | 1612/48710 | 0.806 |
|  | Linear | 0.993 (0.978, 1.007) | / | 0.319 |
| NO_2_ | Q1 | 1 | 1679/57624 |  |
|  | Q2 | 1.024 (0.978, 1.072) | 1738/55346 | 0.309 |
|  | Q3 | 1.021 (0.975, 1.070) | 1715/52484 | 0.375 |
|  | Q4 | 1.056 (1.008, 1.107) | 1605/50676 | 0.023 |
|  | Q5 | 1.079 (1.025, 1.136) | 1472/42772 | 0.004 |
|  | Linear | 1.017 (1.001, 1.033) | / | 0.035 |
| NO_x_ | Q1 | 1 | 1759/58830 |  |
|  | Q2 | 1.003 (0.959, 1.050) | 1708/55192 | 0.882 |
|  | Q3 | 0.980 (0.935, 1.026) | 1659/52126 | 0.382 |
|  | Q4 | 1.030 (0.983, 1.080) | 1532/47542 | 0.216 |
|  | Q5 | 1.019 (0.970, 1.072) | 1551/45212 | 0.449 |
|  | Linear | 1.026 (1.009, 1.044) | / | 0.003 |

Note: Associations were adjusted for batch, centre, age, sex, race, Townsend deprivation index, height, weight, smoking status, alcohol, physical activity, diet, CKD, T2D, cancer, and deprivation. Abbreviations: CI, confidence interval; HR, hazard ratio; PM_2.5_, particular matter with aerodynamic diameter ≤2.5μm; PM_10_, particular matter with an aerodynamic diameter ≤10μm; PM_2.5-10_, particular matter with an aerodynamic diameter between 2.5 and 10μm; NO_2_, nitrogen dioxide; NO_x_, nitrogen oxides; CKD, chronic kidney disease; T2D, Type 2 diabetes.

**Table S10.** **Stratified analysis for different air pollutants concentration with OP risk**

| **Variables** | **Levels** | **eBMD** | | **OP** | | **Fracture** | |
| --- | --- | --- | --- | --- | --- | --- | --- |
|  |  | **Beta (95%CI)** | ***P*** | **HR (95%CI)** | ***P*** | **HR (95%CI)** | ***P*** |
| **NO_x_** | | | | | | | |
| Age | <60 | -0.010 (-0.015, -0.006) | 4.93E-06 | 1.024 (0.990, 1.060) | 0.172 | 1.021 (0.996, 1.046) | 0.096 |
|  | >=60 | -0.009 (-0.014, -0.004) | 2.50E-04 | 1.028 (1.005, 1.051) | 0.015 | 1.017 (0.997, 1.038) | 0.104 |
| Sex | Female | -0.010 (-0.015, -0.006) | 6.60E-06 | 1.026 (1.005, 1.048) | 0.015 | 1.020 (1.001, 1.040) | 0.042 |
|  | Male | -0.010 (-0.015, -0.005) | 1.79E-04 | 1.029 (0.983, 1.077) | 0.219 | 1.016 (0.989, 1.043) | 0.239 |
| BMI | <30 | -0.013 (-0.017, -0.009) | 1.56E-11 | 1.032 (1.011, 1.054) | 0.003 | 1.020 (1.002, 1.038) | 0.031 |
|  | >=30 | -0.002 (-0.008, 0.005) | 0.635 | 1.004 (0.960, 1.049) | 0.872 | 1.015 (0.983, 1.048) | 0.362 |
| Smoking | Never | -0.009 (-0.013, -0.004) | 1.51E-04 | 1.026 (0.999, 1.053) | 0.058 | 1.011 (0.989, 1.033) | 0.344 |
|  | Previous | -0.011 (-0.017, -0.005) | 9.5499E-05 | 1.032 (1.000, 1.064) | 0.047 | 1.031 (1.005, 1.057) | 0.018 |
|  | Current | 0.074 (0.049, 0.098) | 3.68E-09 | 1.026 (0.968, 1.087) | 0.386 | 1.019 (0.973, 1.068) | 0.423 |
| **NO_2_** | | | | | | | |
| Age | <60 | -0.009 (-0.014, -0.004) | 1.48E-04 | 1.016 (0.979, 1.054) | 0.414 | 1.013 (0.987, 1.040) | 0.338 |
|  | >=60 | -0.013 (-0.018, -0.008) | 1.62E-06 | 1.032 (1.007, 1.057) | 0.011 | 1.027 (1.005, 1.050) | 0.016 |
| Sex | Female | -0.005 (-0.010, 0.000) | 0.032 | 1.033 (1.010, 1.057) | 0.004 | 1.025 (1.004, 1.047) | 0.021 |
|  | Male | -0.017 (-0.022, -0.011) | 1.30E-09 | 1.000 (0.951, 1.052) | 0.992 | 1.016 (0.988, 1.046) | 0.265 |
| BMI | <30 | -0.013 (-0.017, -0.009) | 1.23E-10 | 1.037 (1.013, 1.061) | 0.002 | 1.024 (1.005, 1.045) | 0.015 |
|  | >=30 | -0.003 (-0.010, 0.004) | 0.374 | 0.977 (0.931, 1.024) | 0.332 | 1.008 (0.974, 1.043) | 0.652 |
| Smoking | Never | -0.012 (-0.017, -0.007) | 9.23E-07 | 1.036 (1.006, 1.066) | 0.017 | 1.013 (0.989, 1.038) | 0.280 |
|  | Previous | -0.012 (-0.018, -0.006) | 9.17E-05 | 1.030 (0.996, 1.065) | 0.080 | 1.037 (1.009, 1.066) | 0.010 |
|  | Current | 0.074 (0.049, 0.099) | 3.67E-09 | 1.001 (0.940, 1.065) | 0.981 | 1.019 (0.969, 1.071) | 0.461 |
| **PM_10_** | | | | | | | |
| Age | <60 | -0.020 (-0.024, -0.015) | 1.16E-18 | 0.994 (0.960, 1.030) | 0.751 | 0.973 (0.949, 0.998) | 0.032 |
|  | >=60 | -0.020 (-0.025, -0.015) | 1.03E-15 | 1.015 (0.992, 1.039) | 0.208 | 0.994 (0.974, 1.015) | 0.579 |
| Sex | Female | -0.015 (-0.019, -0.010) | 1.06E-10 | 1.010 (0.988, 1.032) | 0.373 | 0.983 (0.964, 1.003) | 0.097 |
|  | Male | -0.026 (-0.031, -0.021) | 1.09E-24 | 1.004 (0.957, 1.054) | 0.861 | 0.990 (0.964, 1.018) | 0.488 |
| BMI | <30 | -0.025 (-0.029, -0.022) | 3.13E-39 | 1.013 (0.992, 1.036) | 0.231 | 0.985 (0.967, 1.003) | 0.100 |
|  | >=30 | -0.006 (-0.013, 0.001) | 0.075 | 0.973 (0.929, 1.018) | 0.231 | 0.983 (0.951, 1.015) | 0.298 |
| Smoking | Never | -0.02 (-0.025, -0.016) | 1.31E-19 | 1.008 (0.981, 1.036) | 0.554 | 0.972 (0.951, 0.994) | 0.013 |
|  | Previous | -0.022 (-0.028, -0.017) | 3.18E-15 | 1.005 (0.973, 1.037) | 0.781 | 1.003 (0.977, 1.029) | 0.832 |
|  | Current | 0.074 (0.049, 0.099) | 3.56E-09 | 1.037 (0.977, 1.100) | 0.237 | 1.000 (0.953, 1.049) | 0.998 |
| **PM_2.5_** | | | | | | | |
| Age | <60 | -0.010 (-0.014, -0.005) | 1.80E-05 | 1.027 (0.992, 1.063) | 0.138 | 1.016 (0.992, 1.042) | 0.198 |
|  | >=60 | -0.006 (-0.011, -0.001) | 0.019 | 1.048 (1.024, 1.072) | 6.86E-05 | 1.010 (0.989, 1.031) | 0.338 |
| Sex | Female | -0.010 (-0.014, -0.005) | 1.93E-05 | 1.040 (1.018, 1.062) | 2.64E-04 | 1.020 (1.000, 1.040) | 0.052 |
|  | Male | -0.007 (-0.012, -0.002) | 0.010 | 1.046 (0.997, 1.097) | 0.067 | 1.001 (0.974, 1.028) | 0.966 |
| BMI | <30 | -0.013 (-0.017, -0.009) | 2.75E-11 | 1.044 (1.022, 1.067) | 7.50E-05 | 1.011 (0.993, 1.030) | 0.231 |
|  | >=30 | 0.004 (-0.003, 0.011) | 0.240 | 1.028 (0.983, 1.075) | 0.231 | 1.018 (0.986, 1.051) | 0.276 |
| Smoking | Never | -0.007 (-0.011, -0.003) | 0.002 | 1.029 (1.002, 1.057) | 0.034 | 1.010 (0.988, 1.033) | 0.379 |
|  | Previous | -0.009 (-0.015, -0.004) | 8.91E-04 | 1.054 (1.021, 1.088) | 0.001 | 1.016 (0.990, 1.042) | 0.243 |
|  | Current | 0.074 (0.049, 0.098) | 3.93E-09 | 1.060 (0.999, 1.124) | 0.054 | 1.021 (0.973, 1.071) | 0.396 |
| **PM_2.5-10_** | | | | | | | |
| Age | <60 | -0.006 (-0.010, -0.002) | 0.004 | 1.012 (0.980, 1.045) | 0.467 | 0.983 (0.961, 1.006) | 0.141 |
|  | >=60 | -0.001 (-0.005, 0.004) | 0.789 | 1.005 (0.984, 1.027) | 0.652 | 1.000 (0.981, 1.019) | 0.973 |
| Sex | Female | -0.002 (-0.006, 0.002) | 0.265 | 1.004 (0.984, 1.023) | 0.710 | 0.995 (0.977, 1.013) | 0.586 |
|  | Male | -0.005 (-0.010, -0.001) | 0.019 | 1.025 (0.980, 1.071) | 0.281 | 0.988 (0.963, 1.013) | 0.341 |
| BMI | <30 | -0.006 (-0.009, -0.003) | 5.55E-04 | 1.005 (0.986, 1.025) | 0.601 | 0.991 (0.975, 1.008) | 0.299 |
|  | >=30 | 0.002 (-0.004, 0.009) | 0.441 | 1.014 (0.973, 1.057) | 0.512 | 0.997 (0.967, 1.027) | 0.829 |
| Smoking | Never | -0.004 (-0.008, 0.000) | 0.060 | 1.000 (0.976, 1.025) | 0.980 | 0.989 (0.969, 1.010) | 0.293 |
|  | Previous | -0.005 (-0.010, 0.000) | 0.041 | 1.004 (0.975, 1.034) | 0.778 | 1.003 (0.979, 1.027) | 0.807 |
|  | Current | 0.074 (0.049, 0.098) | 4.51E-09 | 1.052 (0.998, 1.108) | 0.058 | 0.975 (0.933, 1.020) | 0.272 |

Note: Associations were adjusted for age, sex, genotyped batch, Townsend deprivation index, height, weight, smoking status. Abbreviations: OP, osteoporosis; CI, confidence interval; HR, hazard ratio; PM_2.5_, particular matter with aerodynamic diameter ≤2.5μm; PM_10_, particular matter with an aerodynamic diameter ≤10μm; PM_2.5-10_, particular matter with an aerodynamic diameter between 2.5 and 10μm; NO_2_, nitrogen dioxide; NO_x_, nitrogen oxides; eBMD, estimated bone mineral density; BMI, body mass index.

**Table S11. Stratified analysis for different air pollutants concentration with OP risk (sensitivity analysis)**

| **Variables** | **Levels** | **eBMD** | | **OP** | | **Fracture** | |
| --- | --- | --- | --- | --- | --- | --- | --- |
|  |  | **Beta (95%CI)** | ***P*** | **HR (95%CI)** | ***P*** | **HR (95%CI)** | ***P*** |
| **NO_x_** | | | | | | | |
| Age | <60 | -0.009 (-0.014, -0.005) | 3.73E-05 | 1.020 (0.985, 1.056) | 0.257 | 1.020 (0.995, 1.045) | 0.114 |
|  | >=60 | -0.008 (-0.013, -0.003) | 8.97E-04 | 1.024 (1.001, 1.047) | 0.041 | 1.014 (0.993, 1.034) | 0.196 |
| Sex | Female | -0.010 (-0.014, -0.005) | 2.50E-05 | 1.022 (1.001, 1.044) | 0.042 | 1.017 (0.998, 1.037) | 0.080 |
|  | Male | -0.008 (-0.013, -0.003) | 1.33E-03 | 1.022 (0.975, 1.071) | 0.370 | 1.014 (0.987, 1.041) | 0.311 |
| BMI | <30 | -0.012 (-0.016, -0.008) | 6.81E-10 | 1.029 (1.007, 1.051) | 0.008 | 1.018 (1.000, 1.037) | 0.053 |
|  | >=30 | -0.001 (-0.008, 0.005) | 0.724 | 0.996 (0.952, 1.042) | 0.848 | 1.011 (0.979, 1.044) | 0.510 |
| Smoking | Never | -0.007 (-0.012, -0.003) | 1.12E-03 | 1.018 (0.991, 1.046) | 0.193 | 1.007 (0.985, 1.030) | 0.531 |
|  | Previous | -0.011 (-0.016, -0.005) | 1.79E-04 | 1.030 (0.998, 1.062) | 0.067 | 1.029 (1.003, 1.055) | 0.029 |
|  | Current | 0.056 (0.039, 0.074) | 1.52E-10 | 1.028 (0.970, 1.089) | 0.352 | 1.020 (0.973, 1.068) | 0.416 |
| **NO_2_** | | | | | | | |
| Age | <60 | -0.009 (-0.014, -0.004) | 1.52E-04 | 1.026 (0.988, 1.065) | 0.190 | 1.018 (0.992, 1.046) | 0.182 |
|  | >=60 | -0.012 (-0.018, -0.007) | 3.69E-06 | 1.038 (1.013, 1.064) | 0.003 | 1.029 (1.007, 1.052) | 0.009 |
| Sex | Female | -0.005 (-0.010, -0.001) | 0.030 | 1.041 (1.018, 1.065) | 5.10E-04 | 1.028 (1.007, 1.050) | 0.010 |
|  | Male | -0.016 (-0.021, -0.010) | 8.88E-09 | 0.998 (0.948, 1.051) | 0.941 | 1.021 (0.991, 1.051) | 0.168 |
| BMI | <30 | -0.013 (-0.017, -0.009) | 1.74E-10 | 1.044 (1.020, 1.069) | 2.44E-04 | 1.028 (1.008, 1.049) | 0.005 |
|  | >=30 | -0.003 (-0.010, 0.004) | 0.437 | 0.982 (0.935, 1.031) | 0.468 | 1.010 (0.976, 1.046) | 0.563 |
| Smoking | Never | -0.011 (-0.016, -0.006) | 9.50E-06 | 1.038 (1.008, 1.069) | 0.012 | 1.014 (0.990, 1.039) | 0.240 |
|  | Previous | -0.012 (-0.018, -0.006) | 5.07E-05 | 1.037 (1.002, 1.072) | 0.037 | 1.040 (1.012, 1.069) | 0.006 |
|  | Current | 0.057 (0.039, 0.074) | 1.20E-10 | 1.025 (0.963, 1.092) | 0.435 | 1.033 (0.982, 1.086) | 0.209 |
| **PM_10_** | | | | | | | |
| Age | <60 | -0.020 (-0.025, -0.016) | 1.65E-19 | 1.005 (0.969, 1.041) | 0.806 | 0.978 (0.954, 1.003) | 0.084 |
|  | >=60 | -0.020 (-0.025, -0.015) | 1.21E-15 | 1.021 (0.997, 1.045) | 0.082 | 0.996 (0.975, 1.017) | 0.704 |
| Sex | Female | -0.015 (-0.020, -0.011) | 2.27E-11 | 1.018 (0.996, 1.040) | 0.104 | 0.986 (0.966, 1.005) | 0.153 |
|  | Male | -0.026 (-0.031, -0.021) | 4.13E-24 | 1.003 (0.955, 1.054) | 0.897 | 0.995 (0.968, 1.023) | 0.722 |
| BMI | <30 | -0.026 (-0.030, -0.022) | 7.07E-41 | 1.021 (0.999, 1.044) | 0.059 | 0.988 (0.970, 1.007) | 0.216 |
|  | >=30 | -0.006 (-0.012, 0.001) | 0.091 | 0.979 (0.935, 1.025) | 0.366 | 0.984 (0.952, 1.017) | 0.346 |
| Smoking | Never | -0.020 (-0.024, -0.015) | 3.05E-18 | 1.011 (0.983, 1.039) | 0.438 | 0.973 (0.952, 0.996) | 0.019 |
|  | Previous | -0.023 (-0.029, -0.018) | 2.20E-16 | 1.011 (0.979, 1.044) | 0.503 | 1.005 (0.979, 1.032) | 0.723 |
|  | Current | 0.057 (0.039, 0.074) | 1.45E-10 | 1.064 (1.002, 1.130) | 0.044 | 1.013 (0.965, 1.063) | 0.610 |
| **PM_2.5_** | | | | | | | |
| Age | <60 | -0.008 (-0.013, -0.004) | 2.00E-04 | 1.018 (0.983, 1.054) | 0.326 | 1.013 (0.988, 1.038) | 0.306 |
|  | >=60 | -0.005 (-0.010, 0.000) | 0.059 | 1.039 (1.015, 1.063) | 0.001 | 1.004 (0.984, 1.025) | 0.681 |
| Sex | Female | -0.009 (-0.013, -0.004) | 9.81E-05 | 1.031 (1.009, 1.053) | 0.005 | 1.015 (0.995, 1.035) | 0.149 |
|  | Male | -0.005 (-0.010, 0.000) | 0.059 | 1.035 (0.986, 1.086) | 0.166 | 0.996 (0.969, 1.024) | 0.769 |
| BMI | <30 | -0.011 (-0.015, -0.008) | 4.24E-09 | 1.037 (1.014, 1.059) | 1.15E-03 | 1.007 (0.989, 1.026) | 0.450 |
|  | >=30 | 0.004 (-0.002, 0.011) | 0.195 | 1.014 (0.969, 1.060) | 0.559 | 1.011 (0.979, 1.045) | 0.496 |
| Smoking | Never | -0.006 (-0.010, -0.001) | 0.010 | 1.018 (0.990, 1.045) | 0.206 | 1.004 (0.982, 1.027) | 0.703 |
|  | Previous | -0.009 (-0.014, -0.003) | 0.003 | 1.047 (1.014, 1.080) | 0.005 | 1.011 (0.985, 1.038) | 0.406 |
|  | Current | 0.057 (0.039, 0.074) | 1.45E-10 | 1.056 (0.996, 1.120) | 0.069 | 1.019 (0.972, 1.069) | 0.428 |
| **PM_2.5-10_** | | | | | | | |
| Age | <60 | -0.006 (-0.010, -0.002) | 0.004 | 1.015 (0.983, 1.048) | 0.367 | 0.984 (0.961, 1.006) | 0.159 |
|  | >=60 | -0.001 (-0.005, 0.004) | 0.799 | 1.006 (0.984, 1.027) | 0.612 | 0.999 (0.980, 1.018) | 0.926 |
| Sex | Female | -0.002 (-0.006, 0.002) | 0.244 | 1.005 (0.986, 1.025) | 0.590 | 0.995 (0.977, 1.013) | 0.558 |
|  | Male | -0.005 (-0.010, -0.001) | 0.023 | 1.022 (0.978, 1.068) | 0.325 | 0.989 (0.964, 1.014) | 0.371 |
| BMI | <30 | -0.006 (-0.009, -0.003) | 5.81E-04 | 1.008 (0.988, 1.028) | 0.453 | 0.992 (0.975, 1.008) | 0.327 |
|  | >=30 | 0.002 (-0.004, 0.009) | 0.436 | 1.013 (0.972, 1.056) | 0.543 | 0.995 (0.965, 1.025) | 0.735 |
| Smoking | Never | -0.004 (-0.008, 0.000) | 0.070 | 1.002 (0.977, 1.027) | 0.898 | 0.989 (0.969, 1.009) | 0.281 |
|  | Previous | -0.005 (-0.010, 0.000) | 0.038 | 1.005 (0.976, 1.035) | 0.778 | 1.003 (0.979, 1.027) | 0.831 |
|  | Current | 0.057 (0.039, 0.074) | 1.26E-10 | 1.058 (1.004, 1.114) | 0.035 | 0.977 (0.935, 1.022) | 0.311 |

Note: Associations were adjusted for batch, centre, age, sex, race, Townsend deprivation index, height, weight, smoking status, alcohol, physical activity, diet, CKD, T2D, cancer, and deprivation. Abbreviations: CI, confidence interval; HR, hazard ratio; PM_2.5_, particular matter with aerodynamic diameter ≤2.5μm; PM_10_, particular matter with an aerodynamic diameter ≤10μm; PM_2.5-10_, particular matter with an aerodynamic diameter between 2.5 and 10μm; NO_2_, nitrogen dioxide; NO_x_, nitrogen oxides; CKD, chronic kidney disease; T2D, Type 2 diabetes.

**Table S12. The 95% confidence interval and adjusted hazard ratio for APS with the risk of OP and fracture in the UK Biobank study**

|  |  | **OP** | | | |  | **Fracture** | | | |
| --- | --- | --- | --- | --- | --- | --- | --- | --- | --- | --- |
|  |  | **HR (95% CI)** | **Case/Control** | ***P*** | ***P* for linearity** |  | **HR (95% CI)** | **Case/Control** | ***P*** | ***P* for linearity** |
| APS_1_ | Q1 | 1 | 1530/51893 |  | 8.64E-07 |  | 1 | 2380/51043 |  | 2.29E-04 |
|  | Q2 | 1.027 (0.967, 1.090) | 1552/51870 | 0.388 |  |  | 1.005 (0.956, 1.055) | 2397/51025 | 0.853 |  |
|  | Q3 | 1.049 (0.988, 1.115) | 1590/51832 | 0.117 |  |  | 1.040 (0.990, 1.092) | 2502/50920 | 0.117 |  |
|  | Q4 | 1.094 (1.030, 1.161) | 1721/51701 | 0.004 |  |  | 1.064 (1.013, 1.117) | 2591/50831 | 0.014 |  |
|  | Q5 | 1.140 (1.072, 1.213) | 1816/51606 | 3.17E-05 |  |  | 1.080 (1.026, 1.136) | 2768/50654 | 0.003 |  |
| APS_2_ | Q1 | 1 | 1506/51917 |  | 5.70E-06 |  | 1 | 2388/51035 |  | 1.19E-04 |
|  | Q2 | 1.082 (1.019, 1.149) | 1602/51820 | 0.01 |  |  | 1.004 (0.956, 1.055) | 2365/51057 | 0.869 |  |
|  | Q3 | 1.016 (0.956, 1.080) | 1540/51882 | 0.616 |  |  | 1.042 (0.992, 1.094) | 2509/50913 | 0.101 |  |
|  | Q4 | 1.121 (1.056, 1.190) | 1678/51744 | 1.94E-04 |  |  | 1.071 (1.020, 1.125) | 2603/50819 | 0.006 |  |
|  | Q5 | 1.173 (1.103, 1.247) | 1883/51539 | 3.25E-07 |  |  | 1.077 (1.024, 1.132) | 2773/50649 | 0.004 |  |

Note: Associations were adjusted for age, sex, genotyped batch, Townsend deprivation index, height, weight, smoking status. Abbreviations: OP, osteoporosis; APS, air pollution score; CI, confidence interval; HR, hazard ratio.

**Table S13. The 95% confidence interval and adjusted hazard ratio for APS with the risk of OP and fracture in the UK Biobank study (sensitivity analysis)**

|  |  | **OP** | | | |  | **Fracture** | | | |
| --- | --- | --- | --- | --- | --- | --- | --- | --- | --- | --- |
|  |  | **HR (95% CI)** | **Case/Control** | ***P*** | ***P* for linearity** |  | **HR (95% CI)** | **Case/Control** | ***P*** | ***P* for linearity** |
| APS_1_ | Q1 | 1 | 1530/51893 |  | 2.42E-04 |  | 1 | 2380/51043 |  | 5.08E-04 |
|  | Q2 | 1.012 (0.956, 1.072) | 1552/51870 | 0.685 |  |  | 0.987 (0.942, 1.034) | 2397/51025 | 0.568 |  |
|  | Q3 | 1.052 (0.994, 1.114) | 1590/51832 | 0.080 |  |  | 1.041 (0.994, 1.091) | 2502/50920 | 0.087 |  |
|  | Q4 | 1.067 (1.007, 1.130) | 1721/51701 | 0.028 |  |  | 1.048 (1.000, 1.098) | 2591/50831 | 0.049 |  |
|  | Q5 | 1.093 (1.031, 1.160) | 1816/51606 | 0.003 |  |  | 1.067 (1.017, 1.120) | 2768/50654 | 0.008 |  |
| APS_2_ | Q1 | 1 | 1506/51917 |  | 0.002 |  | 1 | 2388/51035 |  | 5.48E-04 |
|  | Q2 | 1.067 (1.008, 1.129) | 1602/51820 | 0.027 |  |  | 1.002 (0.956, 1.050) | 2365/51057 | 0.943 |  |
|  | Q3 | 1.002 (0.946, 1.062) | 1540/51882 | 0.937 |  |  | 1.030 (0.983, 1.079) | 2509/50913 | 0.209 |  |
|  | Q4 | 1.099 (1.038, 1.164) | 1678/51744 | 1.16E-03 |  |  | 1.070 (1.022, 1.121) | 2603/50819 | 0.004 |  |
|  | Q5 | 1.117 (1.054, 1.184) | 1883/51539 | 2.07E-04 |  |  | 1.068 (1.019, 1.121) | 2773/50649 | 0.006 |  |

Note: Associations were adjusted for batch, centre, age, sex, race, Townsend deprivation index, height, weight, smoking status, alcohol, physical activity, diet, CKD, T2D, cancer, and deprivation. Abbreviations: OP, osteoporosis; APS, air pollution score; CI, confidence interval; HR, hazard ratios; CKD, chronic kidney disease; T2D, Type 2 diabetes.

| **Table S14. OP and fracture risks in the subgroups stratified by genetic risk and air pollution scores (APS_1_) concentrations (sensitivity analysis)** | | | | |
| --- | --- | --- | --- | --- |
| **Traits** | **GRS group** | **APS group** | **HR (95%CI)** | ***P*** |
| OP | High GRS | Q1 | Ref | Ref |
|  |  | Q2 | 1.076 (0.910, 1.273) | 0.390 |
|  |  | Q3 | 1.119 (0.949, 1.320) | 0.180 |
|  |  | Q4 | 1.108 (0.940, 1.307) | 0.223 |
|  |  | Q5 | 1.032 (0.873, 1.219) | 0.714 |
|  | Intermediate GRS | Q1 | 1.326 (1.151, 1.528) | 9.15E-05 |
|  |  | Q2 | 1.350 (1.172, 1.554) | 3.11E-05 |
|  |  | Q3 | 1.360 (1.181, 1.565) | 1.89E-05 |
|  |  | Q4 | 1.384 (1.203, 1.593) | 5.69E-06 |
|  |  | Q5 | 1.412 (1.226, 1.625) | 1.65E-06 |
|  | Low GRS | Q1 | 1.818 (1.564, 2.114) | 8.09E-15 |
|  |  | Q2 | 1.754 (1.508, 2.040) | 3.45E-13 |
|  |  | Q3 | 1.776 (1.528, 2.066) | 8.57E-14 |
|  |  | Q4 | 1.874 (1.614, 2.175) | 1.55E-16 |
|  |  | Q5 | 1.798 (1.548, 2.089) | 1.51E-14 |
| Fracture | High GRS | Q1 | Ref | Ref |
|  |  | Q2 | 0.999 (0.882, 1.131) | 0.988 |
|  |  | Q3 | 1.048 (0.927, 1.184) | 0.453 |
|  |  | Q4 | 1.023 (0.904, 1.157) | 0.716 |
|  |  | Q5 | 1.050 (0.930, 1.187) | 0.431 |
|  | Intermediate GRS | Q1 | 1.103 (0.992, 1.226) | 0.070 |
|  |  | Q2 | 1.086 (0.977, 1.208) | 0.126 |
|  |  | Q3 | 1.139 (1.025, 1.266) | 0.015 |
|  |  | Q4 | 1.216 (1.095, 1.350) | 2.51E-04 |
|  |  | Q5 | 1.161 (1.044, 1.291) | 5.70E-03 |
|  | Low GRS | Q1 | 1.319 (1.173, 1.482) | 3.66E-06 |
|  |  | Q2 | 1.289 (1.146, 1.449) | 2.39E-05 |
|  |  | Q3 | 1.390 (1.238, 1.560) | 2.36E-08 |
|  |  | Q4 | 1.217 (1.081, 1.370) | 1.13E-03 |
|  |  | Q5 | 1.394 (1.243, 1.565) | 1.53E-08 |

Note: Associations were adjusted for batch, centre, age, sex, race, Townsend deprivation index, height, weight, smoking status, alcohol, physical activity, diet, CKD, T2D, cancer, and deprivation. Abbreviations: OP, osteoporosis; APS, air pollution score; CI, confidence interval; CKD, chronic kidney disease; T2D, Type 2 diabetes; GRS, genetic risk score.

**Table S15. OP and fracture risks in the subgroups stratified by genetic risk and air pollution scores (APS_2_) concentrations (sensitivity analysis)**

| **Traits** | **GRS group** | **APS group** | **HR (95%CI)** | ***P*** |
| --- | --- | --- | --- | --- |
| OP | High GRS | Q1 | Ref | Ref |
|  |  | Q2 | 1.081 (0.913, 1.280) | 0.364 |
|  |  | Q3 | 1.112 (0.941, 1.314) | 0.214 |
|  |  | Q4 | 1.059 (0.894, 1.254) | 0.506 |
|  |  | Q5 | 1.164 (0.987, 1.372) | 0.071 |
|  | Intermediate GRS | Q1 | 1.325 (1.147, 1.529) | 1.25E-04 |
|  |  | Q2 | 1.419 (1.231, 1.636) | 1.46E-06 |
|  |  | Q3 | 1.304 (1.129, 1.505) | 3.03E-04 |
|  |  | Q4 | 1.423 (1.235, 1.641) | 1.13E-06 |
|  |  | Q5 | 1.473 (1.278, 1.698) | 9.18E-08 |
|  | Low GRS | Q1 | 1.788 (1.533, 2.084) | 1.13E-13 |
|  |  | Q2 | 1.875 (1.611, 2.183) | 5.08E-16 |
|  |  | Q3 | 1.696 (1.454, 1.979) | 1.82E-11 |
|  |  | Q4 | 1.920 (1.652, 2.233) | 2.15E-17 |
|  |  | Q5 | 1.889 (1.624, 2.196) | 1.37E-16 |
| Fracture | High GRS | Q1 | Ref | Ref |
|  |  | Q2 | 1.036 (0.915, 1.174) | 0.574 |
|  |  | Q3 | 1.061 (0.938, 1.200) | 0.348 |
|  |  | Q4 | 1.055 (0.932, 1.195) | 0.394 |
|  |  | Q5 | 1.085 (0.960, 1.226) | 0.193 |
|  | Intermediate GRS | Q1 | 1.130 (1.015, 1.258) | 0.025 |
|  |  | Q2 | 1.146 (1.030, 1.275) | 0.013 |
|  |  | Q3 | 1.146 (1.030, 1.276) | 0.013 |
|  |  | Q4 | 1.219 (1.096, 1.355) | 2.57E-04 |
|  |  | Q5 | 1.197 (1.076, 1.332) | 9.52E-04 |
|  | Low GRS | Q1 | 1.343 (1.193, 1.511) | 1.01E-06 |
|  |  | Q2 | 1.263 (1.121, 1.424) | 1.32E-04 |
|  |  | Q3 | 1.413 (1.257, 1.587) | 6.39E-09 |
|  |  | Q4 | 1.339 (1.191, 1.506) | 1.12E-06 |
|  |  | Q5 | 1.401 (1.247, 1.575) | 1.40E-08 |

Note: Associations were adjusted for batch, centre, age, sex, race, Townsend deprivation index, height, weight, smoking status, alcohol, physical activity, diet, CKD, T2D, cancer, and deprivation. Abbreviations: OP, osteoporosis; APS, air pollution score; CI, confidence interval; CKD, chronic kidney disease; T2D, Type 2 diabetes; GRS, genetic risk score.
